# FTO promotes clear cell renal cell carcinoma progression via upregulation of PDK1 through an m^6^A dependent pathway

**DOI:** 10.1038/s41420-022-01151-w

**Published:** 2022-08-12

**Authors:** Haixiang Shen, Yufan Ying, Xueyou Ma, Haiyun Xie, Shiming Chen, Jiazhu Sun, Zixiang Liu, Chao Wen, Zitong Yang, Xiao Wang, Mingjie Xu, Jindan Luo, Ben Liu, Jiangfeng Li, Xiangyi Zheng, Liping Xie

**Affiliations:** 1 Department of Urology, the First Affiliated Hospital, Zhejiang University School of Medicine, Hangzhou, 310003 Zhejiang China; 2 Cancer Center, Zhejiang University, Hangzhou, 310058 Zhejiang China

**Keywords:** Renal cell carcinoma, RNA modification

## Abstract

FTO, as an m^6^A mRNA demethylase, is involved in various cancers. However, the role of FTO in clear cell renal cell carcinoma (ccRCC) remains unclear. In the present study, we discovered FTO is upregulated in ccRCC. Functionally, knockdown of FTO significantly impairs the proliferation and migration ability of ccRCC cells. Mechanistically, our data suggest FTO promotes the proliferation and migration of ccRCC through preventing degradation of PDK1 mRNA induced by YTHDF2 in an m^6^A-dependent mechanism. Overall, our results identify the protumorigenic role of FTO through the m^6^A/YTHDF2/PDK1 pathway, which could be a promising therapeutic target for ccRCC.

## Introduction

Renal cell carcinoma (RCC), with clear cell renal cell carcinoma (ccRCC) (~80%) as the primary histological type, is the major malignancy of male genitourinary system following prostate cancer and bladder cancer [1, 2]. Globally, the estimated number of newly diagnosed and deaths of RCC in 2020 was about 431,288 and 179,368, respectively [2]. The routine use of imaging modalities including ultrasound and computed tomography allows an increasing number of RCC patients with small renal masses to be diagnosed at early stage and obtain better prognosis. However, 15–30% of the RCC patients have occurred metastasis when diagnosed initially and about one third of patients with localized RCC eventually develop distant metastasis [3]. Although the mortality of metastatic RCC (mRCC) has declined with the revolution of treatment landscape including targeted therapy and immune checkpoint inhibitors, the prognosis of mRCC still remains dismal [4, 5]. According to the cancer statistic from the American Cancer Society in 2021, 5-year relative survival for patients with mRCC is only about 13% [6]. Therefore, further uncovering the underlying mechanisms involved in the pathogenesis and progression of RCC is critical to advance future treatment paradigm.

N6-methyladenosine (m^6^A), which was first identified in 1970s [7], is the most prevalent and abundant chemical modification in eukaryotic mRNAs [8]. The function and potential molecular mechanism of m^6^A was not unveiled until the availability of specific m^6^A antibody and high-throughput sequencing technologies like RNA m^6^A sequence. In 2011, the function of fat mass and obesity-associated protein (FTO) as an m^6^A mRNA demethylase was firstly identified by Jia et al. [9], suggesting the reversibility of m^6^A modification similar to the epigenetic regulatory mechanisms like DNA and histone modifications. Termed as “writers”, a multicomponent methyltransferase complex composed of METTL3, METTL14 and WTAP catalyzes the installation of m^6^A modification on RNAs, while “erasers” like FTO and ALKBH5 can remove the m^6^A modification, making this posttranscriptional modification process dynamic and reversible. Further, “readers” consisting of YTH domain family (YTHDF1/2/3 and YTHDC1/2), IGF2BP proteins (IGF2BP1/2/3) and heterogeneous nuclear ribonucleoprotein family (HNRNPs) recognize and bind to the m^6^A site to execute the specific functions [10–12]. In particular, emerging evidence suggest m^6^A modification is involved in multiple fundamental bioprocesses, including ultraviolet-caused DNA damage response [13], stem cell differentiation and self-renewal regulation [14], circadian clock [15] and cortical neurogenesis [16]. However, the dysregulation of m^6^A is also involved in a variety of diseases, especially in cancers [17, 18].

FTO, which belongs to the non-heme Fe(II)- and α-KG-dependent dioxygenase AlkB family, is well-known as an obesity-associated gene [19]. In 2011, Jia et al. [9] firstly reported the efficient oxidative demethylation activity of FTO, which prompts renewed interest in the role of FTO in biological functions. In physiological processes, FTO was reported to regulate activity of the dopaminergic midbrain circuitry and formation of adipogenesis in m^6^A-dependent mechanisms [20, 21]. On the other hand, increasing studies indicated that FTO is involved in pathogenesis and progression of various cancers [22–26]. However, the function of FTO in ccRCC still remains to be elucidated.

In the present study, we discovered FTO is upregulated in ccRCC. Furthermore, our data suggest FTO promotes the proliferation and migration of ccRCC through preventing degradation of PDK1 mRNA induced by YTHDF2 in an m^6^A-dependent mechanism. Overall, our results identify the protumorigenic role of FTO through the m^6^A/YTHDF2/PDK1 pathway, which could be a putative therapeutic target for ccRCC.

## Results

### FTO is significantly upregulated in RCC

**Fig. 1:**
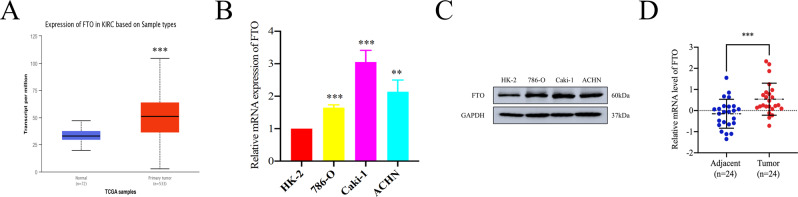
The upregulated expression pattern of FTO in RCC. **A** The transcript levels of FTO in ccRCC vs normal tissues from TCGA samples. KIRC, kidney renal clear cell carcinoma. **B**, **C** The mRNA and protein expression of FTO in HK-2 and RCC cell lines were analyzed by RT-qPCR and Western blot assays, respectively. **D** The mRNA expression of FTO in RCC and the corresponding adjacent tissues. All data are presented as the means ± SDs. **p < 0.01 and ***p < 0.001.

To investigate the role of FTO in RCC, we firstly explored the expression profile of FTO by analyzing the data sets of online databases. From the Oncomine database [27], high expression of FTO in various human cancers including ccRCC was found (Supplementary Fig. 1). Notably, upregulation of FTO in ccRCC and papillary RCC was also observed in UALCAN online databases [28] (Fig. 1A and Supplementary Fig. 2A). Furthermore, the data of GSE16449 downloaded from GEO database also revealed the high expression of FTO in ccRCC (Supplementary Fig. 2B). Meanwhile, we conducted RT-qPCR and Western blot assay to detect the mRNA and protein level of FTO in RCC cell lines. Referred to the normal human proximal tubular epithelial cell line HK-2, the mRNA and protein level were confirmed upregulated in cancer cell lines (Fig. 1B, C and Supplementary Fig. 3A). In addition, the elevated level of FTO in RCC was also confirmed in tumor tissues from our hospital (Fig. 1D and Supplementary Table 4). The results mentioned above suggest that FTO is frequently upregulated in RCC and could be involved in pathogenesis and progression of RCC.

### Knockdown of FTO inhibits the proliferation and migration of ccRCC cells in vitro

**Fig. 2:**
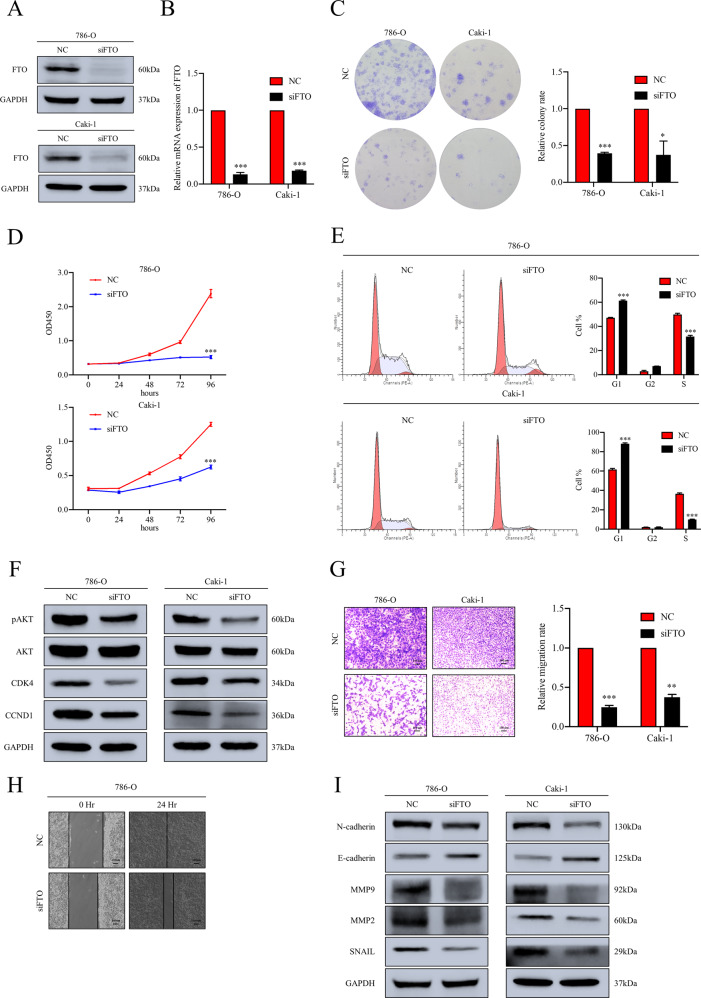
Knockdown of FTO impairs tumor progression of ccRCC in vitro. **A**, **B** The knockdown efficacy of FTO in 786-O and Caki-1 cell lines was confirmed by Western blot and RT-qPCR assays. **C**, **D** Colony formation and CCK-8 assays show the cell proliferation ability decreased after FTO knockdown in 786-O and Caki-1 cell lines. **E** Flow cytometry assay demonstrates knockdown of FTO significantly induced G1 phase arrest in 786-O and Caki-1 cell lines. **F** Western blot assay shows the alterations of CDK4, CCND1 and pAKT (Ser473) in FTO knockdown cell lines. **G** Knockdown of FTO inhibited migration of 786-O and Caki-1 cells detected by trans-well assay. Scale bar 100 μm. **H** Knockdown of FTO retarded the efficacy of healing in 786-O cell line detected by wound healing assay. Scale bar 100 μm. **I** The altered expression of EMT-associated proteins was delineated by Western blot assay in FTO knockdown cell lines. All data are presented as the means ± SDs. *p < 0.05, **p < 0.01 and ***p < 0.001.

Given that ccRCC is the major histopathological type of RCC, this study mainly focused on exploring the role of FTO in ccRCC. We silenced FTO by siRNA to illustrate the potential role of FTO in 786-O and Caki-1 cell lines that were representative of ccRCC. Western blot and RT-qPCR assays were employed to confirm the knockdown efficacy (Fig. 2A, B and Supplementary Fig. 4A). The results of colony formation showed downregulation of FTO obviously impaired the ability of colony formation in ccRCC cell lines (Fig. 2C). CCK-8 assay showed the proliferation of 786-O and Caki-1 cells were inhibited by knockdown of FTO (Fig. 2D). These two experiments suggested that knockdown of FTO impairs the proliferation ability of ccRCC cells. Cell cycle analysis indicated that knockdown of FTO significantly resulted in G1 phase arrest in 786-O and Caki-1 cells (Fig. 2E), which might be responsible for proliferation inhibition by FTO depletion. Furthermore, reduced expression of CDK4 and CCND1 was detected (Fig. 2F and Supplementary Fig. 4B, C), which was consistent with the results of cell cycle analysis. In addition, the alteration of pAKT (Ser473) demonstrated that FTO is implicated in AKT phosphorylation to regulate ccRCC progression (Fig. 2F and Supplementary Fig. 4B, C). We also performed trans-well and wound healing assay to evaluate the influence of FTO depletion on the migration ability in ccRCC cells. The results revealed that downregulation of FTO significantly inhibited cell migration progression of 786-O and Caki-1 cells (Fig. 2G, H). Consistently, Western blot assay showed EMT-associated proteins were suppressed in FTO knockdown cells, indicating that silenced FTO restrained the EMT process of ccRCC cells (Fig. 2I and Supplementary Fig. 4D, E). Overall, we concluded that FTO is involved in proliferation and migration of ccRCC through regulating phosphorylated AKT signalling pathway.

### FTO downregulation restrains the proliferation and migration of ccRCC cells in vivo

**Fig. 3:**
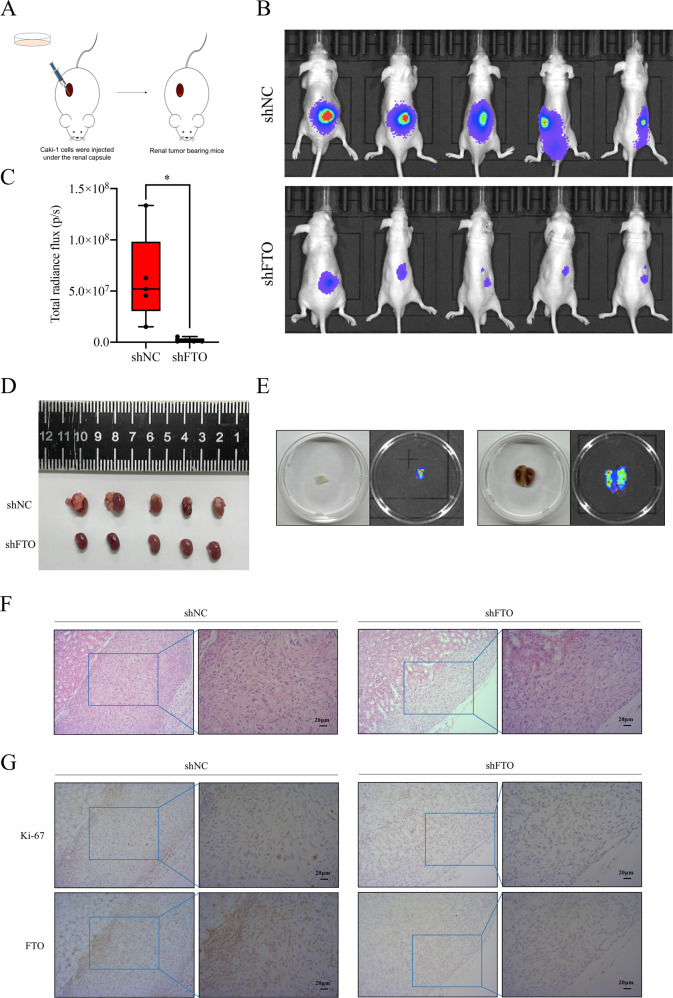
Knockdown of FTO suppresses tumor progression of ccRCC cells in vivo. **A** The schematic illustration for the process of orthotopic transplantation models establishment. **B**, **C** Live imaging revealed the shFTO group presented a weaker luciferase activity when compared to shNC group. **D** Tumor volumes of the two groups. **E** Metastases obtained from shNC group (muscle and lung metastases). **F** H&E staining for the tumor of shNC and shFTO group, respectively. **G** Representative results of IHC for Ki-67 and FTO in the two groups. *p < 0.05.

Furthermore, to explore the function of FTO in vivo, the lentivirus-transfected 786-O and Caki-1 cells with stable knockdown of FTO (shFTO) and negative control (shNC) were established by lentivirus-based shRNA. The knockdown efficacy of FTO with shRNAs was verified by Western blot assay (Supplementary Fig. 5). The orthotopic transplantation models were established as shown in Fig. 3A. Two months later, we measured the relative luciferase activities of the mice through live imaging and the results revealed a significant difference between two groups. The mice of shFTO group showed an obvious drop of the relative luciferase activity when compared to their counterparts (Fig. 3B, C). Then, the mice were sacrificed and the tumor bearing kidneys were anatomized, which indicated knockdown FTO significantly retarded tumor growth (Fig. 3D). Also, metastasis was observed in two mice of shNC group, while no metastasis was found in shFTO group (Fig. 3E and Supplementary Fig. 6). The tumor focus was verified by H&E staining (Fig. 3F). The results of IHC further verified the level of FTO and Ki-67 were consistently depressed in shFTO group compared to shNC group (Fig. 3G). In addition, as a presentative EMT marker widely used in routine IHC, E-cadherin was also detected. As expected, we found the expression of E-cadherin was upregulated in shFTO group when compared to shNC group (Supplementary Fig. 7), which further corroborated the EMT inhibition of FTO knockdown. In summary, all the results confirm that downregulation of FTO impedes the proliferation and migration of ccRCC cells in vivo.

### PDK1 as a downstream target of FTO is regulated via an m^6^A-dependent mechanism

**Fig. 4:**
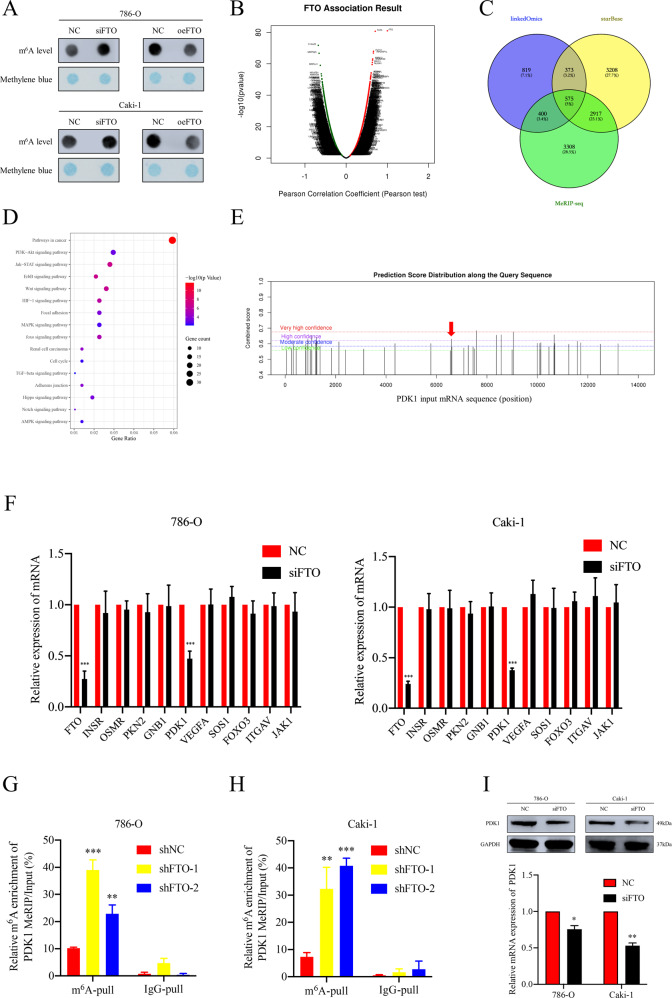
FTO enhances the expression of PDK1 via an m6A-dependent pathway. **A** The alterations of m^6^A level at 200 ng concentration of total RNA with knockdown or overexpression of FTO in 786-O and Caki-1 cells. **B** Genes associated with FTO in LinkedOmics database. **C** Online databases including LinkedOmics, starBase and MeRIP-seq data from previous study were used to find the potential downstream targets of FTO. A total of 575 common genes of the three data sets are displayed by Venn diagram. **D** KEGG pathway enrichment analysis with the potential downstream targets of FTO. **E** The potential m^6^A modification sites of PDK1 mRNA predicted by SRAMP. The peak marked with red arrow was validated by MeRIP assay. **F** RT-qPCR analysis of the potential targets of FTO. **G**, **H** MeRIP-RT-qPCR assay shows the m^6^A level of PDK1 increased after silencing of FTO in 786-O and Caki-1 cells. **I** The protein and mRNA expression levels of PDK1 were detected by Western blot and RT-qPCR after FTO knockdown in 786-O and Caki-1 cells. All data are presented as the means ± SDs. *p < 0.05, **p < 0.01 and ***p < 0.001.

In consideration of the demethylase role of FTO, we speculated that FTO promoted ccRCC progression via an m^6^A-dependent mechanism. To detect the alteration of m^6^A level of RNA in ccRCC cell lines with FTO knockdown, we performed m^6^A RNA dot-blot assay. An increasing tendency of total m^6^A level in FTO-silenced ccRCC cells was found, and the converse was also true when FTO was forced (Fig. 4A). Similar results were observed in RNA m^6^A quantification assay (Supplementary Fig. 8). To identify the potential downstream mRNA targets of FTO, we applied bioinformatics prediction analysis by using online databases. We conducted the co-expression analysis of FTO by using LinkedOmics database [29] and the associated genes were displayed in Fig. 4B. We also obtained the data of FTO-mRNA interactions supported by Clip-seq from starBase database [30]. Combined with the data from two online databases and m^6^A-seq data from the previous study [31], a total of 575 potential target genes of FTO were identified (Fig. 4C). KEGG pathway analysis revealed that these genes were primarily involved in pathways in cancer, focal adhesion, cell cycle and PI3K-AKT signalling pathway (Fig. 4D). Recently, several studies have reported that PI3K-AKT pathway as a key downstream signalling pathway is involved in m^6^A dysregulation in various cancers including endometrial cancer [32], prostate cancer [33] and acute myeloid leukemia (AML) [34]. Therefore, we focused on the genes which were involved in PI3K-AKT pathway and reported in previous studies [35]. From the results of RT-qPCR, among ten potential targets of FTO, only PDK1which is positively associated with FTO was preliminarily verified in FTO-inhibited cells (Fig. 4F). Finally, PDK1 was selected to be the candidate target mRNA of FTO for further research.

We utilized the online tool SRAMP [36] to predict the potential m^6^A modification sites in PDK1 mRNA (Fig. 4E). Me-RIP assay was performed to verified the specific m^6^A modification site interacted with FTO. Compared to control group (shNC group), a notably increased enrichment of m^6^A-modified mRNA of PDK1 by m^6^A specific antibody was observed in shFTO groups (Fig. 4G, H). Also, inhibition in mRNA and protein level of PDK1 was verified after FTO knockdown (Fig. 4I and Supplementary Fig. 4F). Furthermore, overexpression of FTO partially rescued the suppressed expression of PDK1 by siRNA and similar results were discovered in the rescue experiments of phenotypes (Supplementary Fig. 9A–C). Accordingly, PDK1 as the downstream target of FTO was confirmed.

As reported in previous studies, the m^6^A modified site of mRNA is recognized by m^6^A reader proteins which execute the regulation of mRNA metabolism. In terms of m^6^A reader proteins, YTH domain family, especially for YTHDF1 and YTHDF2, are the two mainly validated and well-studied m^6^A readers. Then we used RT-qPCR to explore the influence on PDK1 expression of these two readers with corresponding siRNA for YTHDF1 and YTHDF2 in ccRCC cell lines, respectively. The results showed that the level of PDK1 was upregulated in YTHDF2-silenced cells which was consistent with the knowledge that YTHDF2 intended to exert the degradation of its targets (Supplementary Fig. 10A). However, no significant alteration of PDK1 was observed when YTHDF1 was impaired (Supplementary Fig. 10B). Taken together, YTHDF2 as a potential m^6^A reader was chosen to explore in our study.

**Fig. 5:**
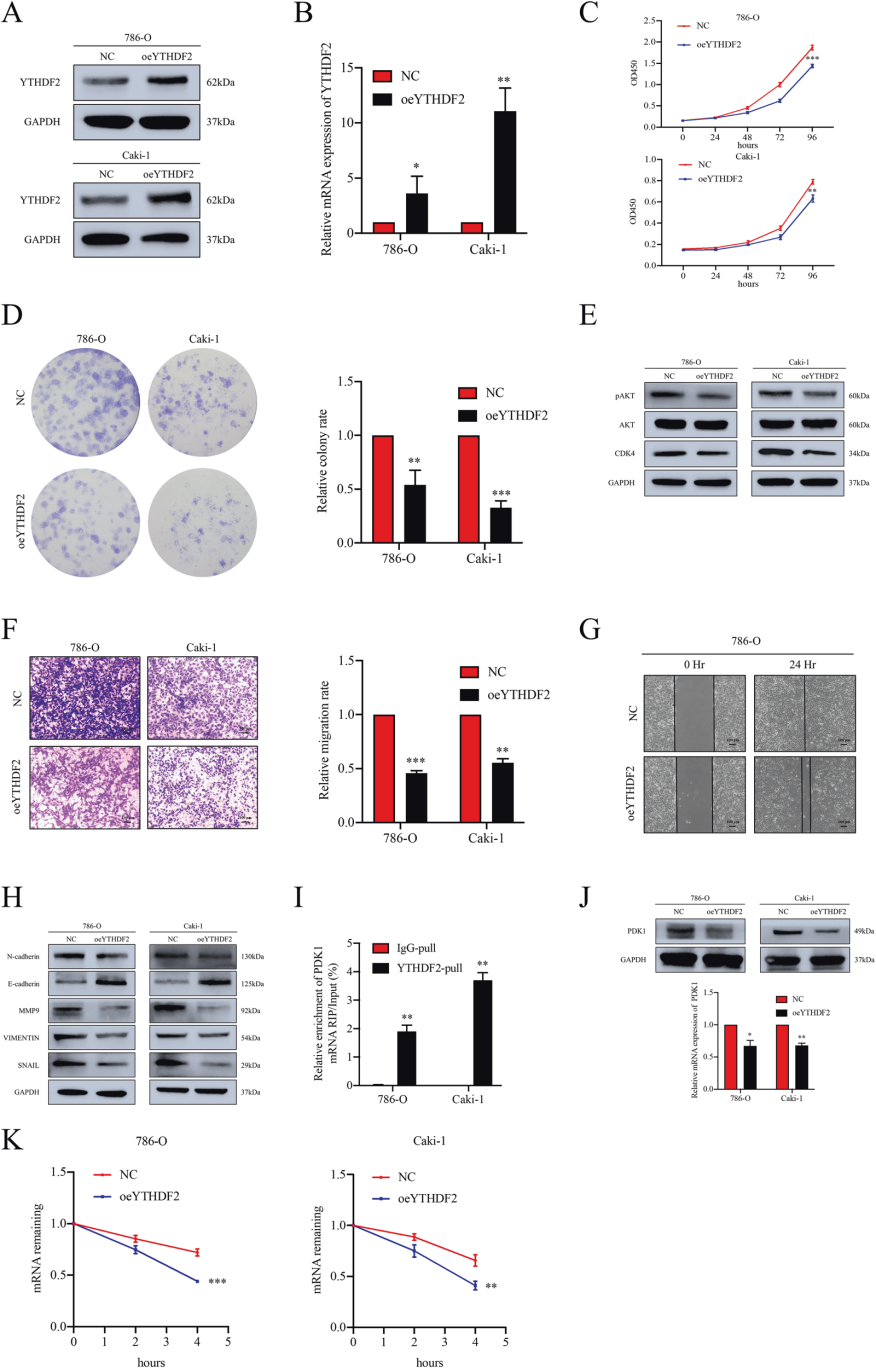
Overexpression of YTHDF2 suppresses tumor progression of ccRCC in vitro. **A**, **B** The overexpression efficacy of YTHDF2 in 786-O and Caki-1 cells was confirmed by Western blot and RT-qPCR assays. **C**, **D** The cell proliferation ability was impaired in YTHDF2 upregulated cells detected by CCK-8 and colony formation assays. **E** Representative Western blots show the altered expression level of CDK4 and pAKT (Ser473) upon the overexpression of YTHDF2. **F** Overexpression of YTHDF2 inhibits migration of 786-O and Caki-1 cells determined by trans-well assay. Scale bar 100 μm. **G** Overexpression of YTHDF2 retarded the efficacy of healing in 786-O cells detected by wound healing assay. Scale bar 100 μm. **H** Significant inhibition of EMT-associated proteins were observed by Western blot assay. **I** RIP-RT-qPCR assay confirmed the binding between YTHDF2 and PDK1 mRNA in 786-O and Caki-1 cells. **J** The protein and mRNA expression levels of PDK1 were detected by Western blot and RT-qPCR after overexpression of YTHDF2 in 786-O and Caki-1 cells. **K** The RNA stability of PDK1 in cells treated with actinomycin D. All data are presented as the means ± SDs. *p < 0.05, **p < 0.01 and ***p < 0.001.

From online databases, downregulated expression of YTHDF2 was discovered and high level of YTHDF2 predicted a better prognosis in ccRCC (Supplementary Fig. 11). Then, we firstly transfected YTHDF2 overexpression plasmid (pYTHDF2) to determine the function of YTHDF2 in ccRCC cells (Fig. 5A, B and Supplementary Fig. 12A). Through a series of functional experiments, we found enforced YTHDF2 suppressed cell proliferation and migration of ccRCC cells (Fig. 5C–H and Supplementary Fig. 12B–E). Subsequently, RIP assay was performed to confirm the integration between YTHDF2 and PDK1. As expected, a notably higher enrichment of PDK1 mRNA by YTHDF2 specific antibody was observed (Fig. 5I and Supplementary Fig. 13). Western blot and RT-qPCR assays further verified the inhibition of PDK1 expression by upregulation of YTHDF2 (Fig. 5J and Supplementary Fig. 12F). In addition, the impaired stability of PDK1 mRNA caused by YTHDF2 overexpression was confirmed (Fig. 5K). Further rescue experiments indicated downregulated YTHDF2 partially restored the suppressed expression of PDK1 and promoted the impaired proliferation and migration progression induced by knockdown of PDK1 (Supplementary Fig. 9D–F). In addition, dual-luciferase reporter assay was conducted to verified the direct interaction between the m^6^A modification site of PDK1 mRNA and YTHDF2. We found the luciferase activity was enforced with YTHDF2 knockdown compared to the control in the wild-type group. However, similar results were not observed in mutated counterpart (Supplementary Fig. 14). In summary, PDK1 is one of the downstream targets regulated by FTO via an m^6^A-dependent mechanism.

### The oncogenic role of PDK1 in ccRCC

**Fig. 6:**
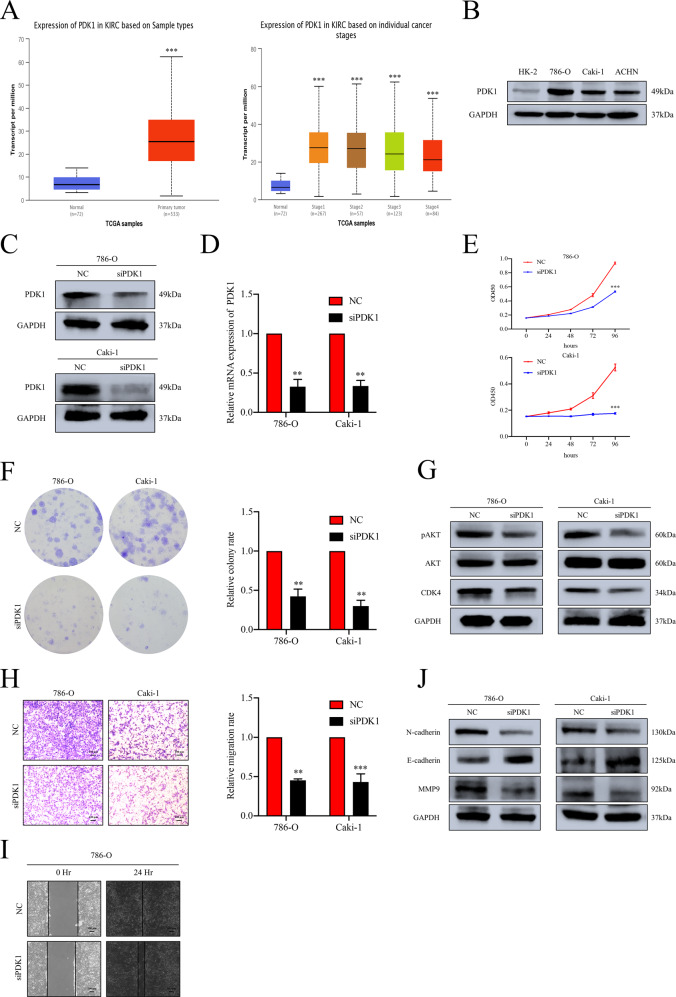
The oncogenic role of PDK1 in ccRCC. **A** The transcript levels of PDK1 in ccRCC vs normal tissues and mRNA expression levels among different tumor stages from TCGA samples. KIRC, kidney renal clear cell carcinoma. **B** The protein expression level of PDK1 was upregulated in RCC cell lines compared to HK-2 analyzed by Western blot assay. **C**, **D** The knockdown efficacy of PDK1 with siRNA in 786-O and Caki-1 cells were verified by Western blot and RT-qPCR assays. **E**, **F** CCK-8 and colony formation assays show the cell proliferation ability impaired after knockdown of PDK1 in 786-O and Caki-1 cells. **G** The alterations of CDK4 and pAKT (Ser473) level were detected by Western blot assay in PDK1 knockdown cells. **H**, **I** Knockdown of PDK1 retarded the progression of migration and wound healing in 786-O and Caki-1 cells detected by trans-well and wound healing assay. Scale bar 100 μm. **J** The altered expression of EMT-associated proteins was detected by Western blot assay upon the knockdown of PDK1. All data are presented as the means ± SDs. **p < 0.01 and ***p < 0.001.

**Fig. 7:**
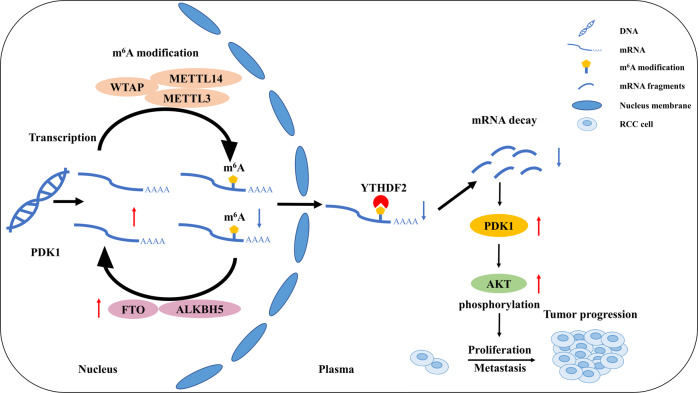
Schematic diagram of the proposed mechanism in this study. In brief, upregulated of FTO induces an elimination of m^6^A modification level on PDK1 mRNA, which is specifically recognized and degraded by m^6^A reader YTHDF2. Accumulated PDK1 promotes tumor progression via AKT phosphorylation in ccRCC eventually.

From UALCAN online database [28], we firstly obtained the upregulated expression profile of PDK1 in ccRCC when compared to normal tisues (Fig. 6A). The high expression profile of PDK1 was also confirmed in RCC cell lines by Western blot, which suggested that PDK1 might be involved in the tumorigenesis and progression of ccRCC (Fig. 6B and Supplementary Fig. 3B). To elucidate the role of PDK1, the expression of PDK1 was suppressed by specific siRNA (Fig. 6C, D and Supplementary Fig. 15A). Functionally, PDK1 knockdown obviously impaired the proliferation of ccRCC cells (Fig. 6E, F). Representative Western blots showed a similar alteration of indicated proteins (Fig. 6G and Supplementary Fig. 15B, C). The retarded migration progression induced by depletion of PDK1 was revealed by trans-well and wound healing assays (Fig. 6H, I). Western blot assay demonstrated the corresponding alteration of proteins involved in EMT progression (Fig. 6J and Supplementary Fig. 15D, E). Interestingly, similar alteration of pAKT which was consistent with the changes after knockdown of FTO was also observed (Fig. 6G). These evidences above verified the oncogenic role of PDK1 in ccRCC. Taken all together, we concluded that FTO overexpression decreases the m^6^A modification level of PDK1, which prevents degradation of PDK1 mRNA induced by YTHDF2, and then enforced PDK1 promotes progression of ccRCC via AKT phosphorylation (Fig. 7).

## Discussion

Currently, increasing studies have reported the critical role of m^6^A modification in multiple physiological and pathological processes, especially in various tumors [18]. However, the role of m^6^A in the tumorigenesis and progression of ccRCC remains to be investigated. In the present study, we revealed the oncogenic role of FTO in ccRCC. Briefly, we firstly found FTO was upregulated in cell lines and tumor tissues of ccRCC. Functionally, knockdown of FTO impaired the proliferation and migration ability of ccRCC cells in vitro and in vivo. Mechanically, upregulated FTO promotes ccRCC through the regulation of PDK1 mRNA stability in an m^6^A-dependent and YTHDF2-associated pathway. FTO-enforced PDK1 then activated the phosphorylation of AKT and promoted the progression of ccRCC.

FTO, as a member of the non-heme Fe(II)- and α-KG-dependent dioxygenase AlkB family, was reported as an obesity-associated gene in previous studies [19]. Recently, renewed attention has been focused on the function of FTO since Jia and colleagues redefined FTO as an m^6^A demethylase in 2011 [9].

Subsequently, emerging evidence demonstrated that FTO is involved in a variety of cancers via an m^6^A-dependent mechanism. Li et al. reported that FTO promoted the progression of AML by facilitating the transformation of leukemic oncogene-mediated cell and leukemogenesis and repressing the response of APL cells to ATRA treatment through down-regulating ASB2 and RARA expression in an m^6^A-dependent pathway [22]. In melanoma, Yang and colleagues found FTO played a crucial oncogenic role in promoting tumorigenesis and resistance to interferon gamma and anti-PD-1 treatment [25]. Furthermore, Zhang et al. indicated that FTO promoted endometrial cancer metastasis via m^6^A-HOXB13-WNT pathway [37]. Similar results were reported in other malignancies [23, 24, 38]. In the present study, we uncovered the oncogenic role of FTO in ccRCC. Mechanically, FTO reduced the epigenetic m^6^A modification level of PDK1 mRNA, preventing PDK1 from degrading by YTHDF2. Further, accumulated PDK1 activated phosphorylation of AKT and eventually promoted ccRCC progression.

During the process of the m^6^A modification, readers are the key component which exert corresponding function to regulate RNA metabolism. As the first discovered and well-studied m^6^A reader protein, YTHDF2 binds to the specific m^6^A site and induces the degradation of RNA [39, 40]. Conversely, YTHDF1 recognizes the m^6^A modification and promotes mRNA translation [41]. Cooperating with YTHDF2 or YTHDF1, YTHDF3 acts as a promotor to enhance the function of RNA degradation or mRNA translation, respectively [42]. When m^6^A-modified pre-mRNAs recognized by YTHDC1, splicing factor SRSF3 is recruited while SRSF10 is blocked to nuclear speckles, which accelerates exon inclusion, splicing and transport of mRNA from nucleus to cytoplasm [43, 44]. For YTHDC2, it exerts the function of enhancing the translation while reducing the abundance of target mRNAs [45]. The members of IGF2BPs (IGF2BP1/2/3) play a similar role as YTHDF1 when interact with m^6^A-modified mRNA [46]. Furthermore, a new mechanism termed as “the m^6^A-switch” was pioneered by Liu et al., which illustrated how m^6^A reader proteins approach to targeted RNAs [47, 48]. In our study, we discovered that knockdown of FTO increased the m^6^A level but decreased the expression of PDK1, suggesting YTHDF2 probably acted as an m^6^A reader in this process. Subsequent RIP assay, dual-luciferase reporter assay and rescue experiments validated YTHDF2 was involved in the mechanism as a reader protein.

Pyruvate dehydrogenase kinase 1 (PDK1), which is localized on the chromosome 2q31.1, is mainly involved in glycolysis by inactivating the pyruvate dehydrogenase complex. Recently, PDK1 was reported as an oncogene in various cancers. Siu and colleagues found the expression of PDK1 was high in ovarian cancer. And the patients with increased PDK1 were prone to harbor metastasis and suffered from reduced chemosensitivity and poor overall survival. Further, they demonstrated that PDK1 regulated tumor-mesothelial adhesion, invasion, and angiogenesis via α5β1 integrin and JNK/IL-8 signaling pathways and eventually promoted ovarian cancer metastasis [49]. In melanoma, PDK1 was revealed to facilitate resistance of targeted BRAF inhibition through mediating oncogene-induced senescence induced by BRAF^V600E^ [50]. Also, evidenced by Chen et al., PDK1-depend aerobic glycolysis was required for proliferation and survival of leukemia KG1a cells and lung cancer [51]. In our study, consistent with previous studies, we found PDK1 was upregulated and involved in the progression of ccRCC. With bioinformatics analysis and validation by a series of assays like Me-RIP, PDK1 was confirmed as a downstream target of FTO. Mechanically, enhanced expression of PDK1 induced by FTO/YTHDF2/m^6^A pathway facilitated ccRCC progression via activating AKT phosphorylation.

Given the steadily increasing incidence and poor prognosis of ccRCC, especially for patients with metastasis, it is of significant importance to understand the molecular mechanisms of tumorigenesis for ccRCC. With the understanding of m^6^A disregulation involved in several cancers, several small-molecule inhibitors of m^6^A demethylases and methyltransferases have been available. In 2012, Chen et al. first identified rhein as the most potent inhibitor of FTO which suppresses m^6^A demethylation activity through competitively binding to catalytic site of FTO [52]. The good inhibitory activity on m^6^A demethylation of rhein was also confirmed in vivo which can significantly inhibit the progression of breast tumor and AML [26, 53]. FB23-2, a small-molecule compound developed by Huang et al., exerts a similar tumor-suppressing function in AML by inhibiting FTO [54]. As for inhibitors, exploring small-molecule compounds which are targeted for methyltransferases has been proposed as another strategy. As reported in a recent study, AML growth retardation and prolonged survival was observed in mouse models of AML treated with a highly potent and selective catalytic inhibitor of METTL3 named STM2457 [55]. Collectively, increasing studies reveal the development of small-molecule inhibitors targeted for m^6^A regulators as a potential and promising therapeutic strategy against cancers. In our study, we uncovered the vital role of FTO/YTHDF2/PDK1/m^6^A-dependent axis involved in the tumorigenesis and progression of ccRCC, which might provide potential therapeutic targets for ccRCC in the future.

## Conclusions

In summary, we found upregulated FTO promotes proliferation and migration of ccRCC through the regulation of PDK1 mRNA stability in an m^6^A-dependent and YTHDF2-associated pathway. FTO-enhanced PDK1 facilitates ccRCC progression via activating AKT phosphorylation. Altogether, our study demonstrates FTO acts as a crucial oncogene in ccRCC through the m^6^A/YTHDF2/PDK1 pathway, which might be helpful to understand the molecular mechanism in ccRCC progression and unveil potential promising targets for therapy.

## Materials and methods

### Cell lines and cell culture

HK-2, 786-O, Caki-1 and ACHN were purchased from the Cell Bank of the Chinese Academy of Sciences (Shanghai, China). The HK-2 cell line and RCC cell lines were cultured in cell incubator (ThermoFisher, USA) under the 5% CO2 humidified atmosphere at 37 °C, with K-SFM (Gibco) and RPMI-1640 (BI) medium with 10% heat-inactivated foetal bovine serum (BI), respectively.

### Tissue samples

From January to October 2013, we obtained 24 paired specimens of tumor and adjacent normal tissues from RCC patients treated with radical nephrectomy in our hospital [56]. Written informed consents of patients were acquired.

### Reagents and transfection

We used Polyplus transfection® reagent (Proteintech, Chicago, USA) to perform transfection experiments in accordance with the operation manual. The specific steps were performed as previously depicted [57]. All corresponding targeted sequences of siRNAs are listed in Supplementary Table 1.

### Lentiviruses and infection

Stable knockdown cell lines were established by using the vector GV344 purchasing from Genechem with corresponding lentivirus-based short-hairpin RNAs. ccRCC cell lines were infected by the lentivirus and the stable knockdown cells were screened out with puromycin following the manufacturer’s protocols. All the associated shRNAs sequences are summarized in Supplementary Table 1.

### RNA extraction and RT-qPCR

The detailed procedure was conducted in accord with previous study [57]. All associated primers were listed in Supplementary Table 2.

### Western blot assay

The specific steps of the experiment were performed as previously reported [57]. All the associated primary antibodies were summarized in Supplementary Table 3.

### Cell counting kit-8 (CCK-8) assay

Transfected cells were seeded at a density of 2000 cells per well in 96-well plates with 200 μL fresh culture medium and cultured for 0, 24, 48, 72 and 96 hours. Incubated with CCK-8 reagent (#CK04, Dojindo Laboratories, Japan) at 37 °C for 2 hours, the microplate reader (BioTek, USA) was used to measure the absorbance of solution at 450 nm.

### Colony formation assay

A density of 500 transfected cells per well were seeded in 6-well plates and cultured for 7-12 days. Fixed with absolute methanol and then stained with 0.3% crystal violet, the number of colony were analyzed.

### Trans-well assay

Trans-well assay was conducted to assess the migration ability of transfected cells. The cell suspension containing 5 × 10^4^ 786-O or 7 × 10^4^ Caki-1 cells diluted with 200 μL serum-free medium was added into the trans-well chambers (#MCEP24H48, Merck Millipore) located in a 24-well plate. 800 μL culture medium with 10% foetal bovine serum was filled into the compartment between the well and trans-well chamber. After incubation at 37 °C for 24 h, the migrated cells were fixed with absolute methanol and then stained with 0.3% crystal violet. After wiping off the cells on the upper surface of chamber with a cotton swab, the images of migrated cells were taken by phase-contrast microscopy (IX71, Olympus) under a 10× objective lens.

### Wound-healing assay

Treated cells with 100 μL standard medium were seeded into each well of silicone culture insert which was fixed in a 6-well plate. Incubated overnight at 37 °C and 5% CO_2_, a confluent monolayer of cells was achieved. After removing the insert, cells were cultured in serum-free medium. Phase-contrast microscopy with a 10× objective lens was used to monitor the cellular migration process.

### Cell cycle analysis

The specific steps of cell cycle analysis were performed as previously depicted [58].

### RNA m^6^A dot blot assay

Total RNAs extracted from cell lines were adjusted to the concentration of 50 ng/μL in 36 μL RNase-free water and then mixed with 108 μL RNA incubation buffer. Further incubation at 65 °C was conducted to remove secondary structure. 200 ng pre-treated RNA was added onto the Amersham Hybond-N + membrane (#RPN303B, GE health, Chicago, USA) through the Bio-Dot Apparatus (Bio-Rad, USA). Thereafter, RNAs on the membrane were UV cross-linked for five minutes and incubated in 0.02% methylene blue (#M9140-25G, Sigma-Aldrich, USA). The scanning of dot methylene blue on the membrane was obtained to ensure the equal amount of RNAs added. Subsequently, the membrane was blocked in 5% non-fat milk for one hour and incubated with m^6^A-specific antibody overnight. The membrane was washed with PBST for three times in total thirty minutes and then incubated with corresponding secondary antibody. The membrane implemented with ECL Kits (Biosharp, China) was finally visualized by the imaging system.

### m^6^A-RNA immunoprecipitation assay

The m^6^A-RNA immunoprecipitation (MeRIP) assay was performed as our previous study [56]. The primers for RT-qPCR analysis were listed in Supplementary Table 2.

### RNA-binding protein immunoprecipitation assay

RNA-binding protein immunoprecipitation (RIP) assay was performed with Magna RIP™ RNA-Binding Protein Immunoprecipitation Kit (#17-701, Merck Millipore) following the operation manual. Briefly, the cells cultured in 15-cm plates were lysed and collected without the supernatant after the centrifugation. The precipitate was further processed for immunoprecipitation. The proteins and RNAs were extracted and purified for Western blot and RT-qPCR.

### Dual-luciferase reporter assay

For dual-luciferase reporter assay, the wild-type and mutated sequence of m^6^A site were designed and synthesized (Supplementary Table 2). The sequences constructed were cloned into pmirGlo luciferase expression vector (Promega, Madison, USA) which were further verified by DNA sequencing. All the specific operations were performed as our previous study [33].

### Animal experiments

Orthotopic transplantation models with male BALB/c nude mice (four weeks old) were performed as reported in previous study [59]. All animal operations were approved and conducted in accord with institutional guidelines of the First Affiliated Hospital, Zhejiang University School of Medicine.

### Immunohistochemistry

The xenograft tumor specimens were anatomized from mice. Immunohistochemistry (IHC) analysis was applied to determine the expression level of associated proteins. The detailed operating procedures were consistent with the previous study [57].

### Bioinformatics analysis

Several online databases were used to performed bioinformatics analysis. The UALCAN (http://ualcan.path.uab.edu) and Oncomine (https://www.oncomine.org/resource/login.html) databases were utilized to analyze the expression profile of focused genes in our study. The data of GSE16449 downloaded from GEO database (https://www.ncbi.nlm.nih.gov/geo/) were analyzed to further validate the FTO expression pattern in ccRCC. The combination of LinkedOmics (http://www.linkedomics.org/), starBase (http://starbase.sysu.edu.cn/) and SRAMP (http://www.cuilab.cn/sramp) were used for prediction of potential target genes. Venny 2.1 (https://bioinfogp.cnb.csic.es/tools/venny/index.html) was used for drawing Venn diagram. KEGG pathway analysis was conducted by Metascape database (http://metascape.org/).

### Statistical analysis

All the data were presented as the mean ± SD. GraphPad Prism version 8.4.3 was used for all statistical analyses. A two-tailed Student’s *t* test was applied in comparison between groups and *p* < 0.05 was defined as statistical significance.

## Supplementary information

Supplementary materials and methods

Supplementary Figure Legends

Supplementary Figure 1

Supplementary Figure 2

Supplementary Figure 3

Supplementary Figure 4

Supplementary Figure 5

Supplementary Figure 6

Supplementary Figure 7

Supplementary Figure 8

Supplementary Figure 9

Supplementary Figure 10

Supplementary Figure 11

Supplementary Figure 12

Supplementary Figure 13

Supplementary Figure 14

Supplementary Figure 15

qPCR raw data

WB densities

Supplementary Tables

Original full length western blots

## Data Availability

All data generated or analyzed during this study are included in this article and supplementary files.

## References

[1] Escudier B, Porta C, Schmidinger M, Rioux-Leclercq N, Bex A, Khoo V (2019). Renal cell carcinoma: ESMO Clinical Practice Guidelines for diagnosis, treatment and follow-up. Ann Oncol: Off J Eur Soc Med Oncol.

[2] Sung H, Ferlay J, Siegel RL, Laversanne M, Soerjomataram I, Jemal A (2021). Global Cancer Statistics 2020: GLOBOCAN Estimates of Incidence and Mortality Worldwide for 36 Cancers in 185 Countries. CA: a cancer J clinicians.

[3] Posadas EM, Limvorasak S, Figlin RA (2017). Targeted therapies for renal cell carcinoma. Nat Rev Nephrol.

[4] Ljungberg B, Campbell SC, Choi HY, Jacqmin D, Lee JE, Weikert S (2011). The epidemiology of renal cell carcinoma. Eur Urol.

[5] Choueiri TK, Motzer RJ (2017). Systemic therapy for metastatic renal-cell carcinoma. N Engl J Med.

[6] Siegel RL, Miller KD, Fuchs HE, Jemal A (2021). Cancer Statistics, 2021. CA: a cancer J clinicians.

[7] Adams JM, Cory S (1975). Modified nucleosides and bizarre 5’-termini in mouse myeloma mRNA. Nature.

[8] Saletore Y, Meyer K, Korlach J, Vilfan ID, Jaffrey S, Mason CE (2012). The birth of the Epitranscriptome: deciphering the function of RNA modifications. Genome Biol.

[9] Jia G, Fu Y, Zhao X, Dai Q, Zheng G, Yang Y (2011). N6-methyladenosine in nuclear RNA is a major substrate of the obesity-associated FTO. Nat Chem Biol.

[10] Frye M, Harada BT, Behm M, He C (2018). RNA modifications modulate gene expression during development. Sci (N. Y, NY).

[11] Zaccara S, Ries RJ, Jaffrey SR (2019). Reading, writing and erasing mRNA methylation. Nat Rev Mol cell Biol.

[12] Shi H, Wei J, He C (2019). Where, when, and how: context-dependent functions of RNA methylation writers, readers, and erasers. Mol cell.

[13] Xiang Y, Laurent B, Hsu CH, Nachtergaele S, Lu Z, Sheng W (2017). RNA m(6)A methylation regulates the ultraviolet-induced DNA damage response. Nature.

[14] Zhang C, Chen Y, Sun B, Wang L, Yang Y, Ma D (2017). m(6)A modulates haematopoietic stem and progenitor cell specification. Nature.

[15] Fustin JM, Kojima R, Itoh K, Chang HY, Ye S, Zhuang B (2018). Two Ck1δ transcripts regulated by m6A methylation code for two antagonistic kinases in the control of the circadian clock. Proc Natl Acad Sci USA.

[16] Yoon KJ, Ringeling FR, Vissers C, Jacob F, Pokrass M, Jimenez-Cyrus D (2017). Temporal Control of Mammalian Cortical Neurogenesis by m(6)A Methylation. Cell.

[17] Yang Y, Shen F, Huang W, Qin S, Huang JT, Sergi C (2019). Glucose is involved in the dynamic regulation of m6A in patients with type 2 Diabetes. J Clin Endocrinol Metab.

[18] Lan Q, Liu PY, Haase J, Bell JL, Hüttelmaier S, Liu T (2019). The critical role of RNA m(6)A methylation in cancer. Cancer Res.

[19] Church C, Moir L, McMurray F, Girard C, Banks GT, Teboul L (2010). Overexpression of Fto leads to increased food intake and results in obesity. Nat Genet.

[20] Hess ME, Hess S, Meyer KD, Verhagen LA, Koch L, Brönneke HS (2013). The fat mass and obesity associated gene (Fto) regulates activity of the dopaminergic midbrain circuitry. Nat Neurosci.

[21] Zhao X, Yang Y, Sun BF, Shi Y, Yang X, Xiao W (2014). FTO-dependent demethylation of N6-methyladenosine regulates mRNA splicing and is required for adipogenesis. Cell Res.

[22] Li Z, Weng H, Su R, Weng X, Zuo Z, Li C (2017). FTO plays an oncogenic role in acute myeloid leukemia as a N(6)-methyladenosine RNA demethylase. Cancer cell.

[23] Cui Q, Shi H, Ye P, Li L, Qu Q, Sun G (2017). m(6)A RNA methylation regulates the self-renewal and tumorigenesis of glioblastoma stem cells. Cell Rep.

[24] Zhou S, Bai ZL, Xia D, Zhao ZJ, Zhao R, Wang YY (2018). FTO regulates the chemo-radiotherapy resistance of cervical squamous cell carcinoma (CSCC) by targeting β-catenin through mRNA demethylation. Mol carcinogenesis.

[25] Yang S, Wei J, Cui YH, Park G, Shah P, Deng Y (2019). m(6)A mRNA demethylase FTO regulates melanoma tumorigenicity and response to anti-PD-1 blockade. Nat Commun.

[26] Niu Y, Lin Z, Wan A, Chen H, Liang H, Sun L (2019). RNA N6-methyladenosine demethylase FTO promotes breast tumor progression through inhibiting BNIP3. Mol cancer.

[27] Rhodes DR, Kalyana-Sundaram S, Mahavisno V, Varambally R, Yu J, Briggs BB (2007). Oncomine 3.0: genes, pathways, and networks in a collection of 18,000 cancer gene expression profiles. Neoplasia (N. Y, NY).

[28] Chandrashekar DS, Bashel B, Balasubramanya SAH, Creighton CJ, Ponce-Rodriguez I, Chakravarthi B (2017). UALCAN: A Portal for Facilitating Tumor Subgroup Gene Expression and Survival Analyses. Neoplasia (N. Y, NY).

[29] Vasaikar SV, Straub P, Wang J, Zhang B (2018). LinkedOmics: analyzing multi-omics data within and across 32 cancer types. Nucleic acids Res.

[30] Li JH, Liu S, Zhou H, Qu LH, Yang JH (2014). starBase v2.0: decoding miRNA-ceRNA, miRNA-ncRNA and protein-RNA interaction networks from large-scale CLIP-Seq data. Nucleic acids Res.

[31] Dominissini D, Moshitch-Moshkovitz S, Schwartz S, Salmon-Divon M, Ungar L, Osenberg S (2012). Topology of the human and mouse m6A RNA methylomes revealed by m6A-seq. Nature.

[32] Liu J, Eckert MA, Harada BT, Liu SM, Lu Z, Yu K (2018). m(6)A mRNA methylation regulates AKT activity to promote the proliferation and tumorigenicity of endometrial cancer. Nat cell Biol.

[33] Li J, Xie H, Ying Y, Chen H, Yan H, He L (2020). YTHDF2 mediates the mRNA degradation of the tumor suppressors to induce AKT phosphorylation in N6-methyladenosine-dependent way in prostate cancer. Mol cancer.

[34] Vu LP, Pickering BF, Cheng Y, Zaccara S, Nguyen D, Minuesa G (2017). The N(6)-methyladenosine (m(6)A)-forming enzyme METTL3 controls myeloid differentiation of normal hematopoietic and leukemia cells. Nat Med.

[35] Li XD, Wang MJ, Zheng JL, Wu YH, Wang X, Jiang XB (2021). Long noncoding RNA just proximal to X-inactive specific transcript facilitates aerobic glycolysis and temozolomide chemoresistance by promoting stability of PDK1 mRNA in an m6A-dependent manner in glioblastoma multiforme cells. Cancer Sci.

[36] Zhou Y, Zeng P, Li YH, Zhang Z, Cui Q (2016). SRAMP: prediction of mammalian N6-methyladenosine (m6A) sites based on sequence-derived features. Nucleic acids Res.

[37] Zhang L, Wan Y, Zhang Z, Jiang Y, Lang J, Cheng W (2021). FTO demethylates m6A modifications in HOXB13 mRNA and promotes endometrial cancer metastasis by activating the WNT signalling pathway. RNA Biol.

[38] Li J, Han Y, Zhang H, Qian Z, Jia W, Gao Y (2019). The m6A demethylase FTO promotes the growth of lung cancer cells by regulating the m6A level of USP7 mRNA. Biochemical biophysical Res Commun.

[39] Wang X, Lu Z, Gomez A, Hon GC, Yue Y, Han D (2014). N6-methyladenosine-dependent regulation of messenger RNA stability. Nature.

[40] Du H, Zhao Y, He J, Zhang Y, Xi H, Liu M (2016). YTHDF2 destabilizes m(6)A-containing RNA through direct recruitment of the CCR4-NOT deadenylase complex. Nat Commun.

[41] Wang X, Zhao BS, Roundtree IA, Lu Z, Han D, Ma H (2015). N(6)-methyladenosine modulates messenger RNA translation efficiency. Cell.

[42] Shi H, Wang X, Lu Z, Zhao BS, Ma H, Hsu PJ (2017). YTHDF3 facilitates translation and decay of N(6)-methyladenosine-modified RNA. Cell Res.

[43] Xiao W, Adhikari S, Dahal U, Chen YS, Hao YJ, Sun BF (2016). Nuclear m(6)A reader YTHDC1 regulates mRNA splicing. Mol cell.

[44] Roundtree IA, Luo GZ, Zhang Z, Wang X, Zhou T, Cui Y, et al. YTHDC1 mediates nuclear export of N(6)-methyladenosine methylated mRNAs. eLife 2017;6:e31311. 10.7554/eLife.31311 PMC5648532 28984244

[45] Hsu PJ, Zhu Y, Ma H, Guo Y, Shi X, Liu Y (2017). Ythdc2 is an N(6)-methyladenosine binding protein that regulates mammalian spermatogenesis. Cell Res.

[46] Huang H, Weng H, Sun W, Qin X, Shi H, Wu H (2018). Recognition of RNA N(6)-methyladenosine by IGF2BP proteins enhances mRNA stability and translation. Nat cell Biol.

[47] Liu N, Dai Q, Zheng G, He C, Parisien M, Pan T (2015). N(6)-methyladenosine-dependent RNA structural switches regulate RNA-protein interactions. Nature.

[48] Liu N, Zhou KI, Parisien M, Dai Q, Diatchenko L, Pan T (2017). N6-methyladenosine alters RNA structure to regulate binding of a low-complexity protein. Nucleic acids Res.

[49] Siu MKY, Jiang YX, Wang JJ, Leung THY, Ngu SF, Cheung ANY (2020). PDK1 promotes ovarian cancer metastasis by modulating tumor-mesothelial adhesion, invasion, and angiogenesis via α5β1 integrin and JNK/IL-8 signaling. Oncogenesis.

[50] Kaplon J, Zheng L, Meissl K, Chaneton B, Selivanov VA, Mackay G (2013). A key role for mitochondrial gatekeeper pyruvate dehydrogenase in oncogene-induced senescence. Nature.

[51] Hitosugi T, Fan J, Chung TW, Lythgoe K, Wang X, Xie J (2011). Tyrosine phosphorylation of mitochondrial pyruvate dehydrogenase kinase 1 is important for cancer metabolism. Mol cell.

[52] Chen B, Ye F, Yu L, Jia G, Huang X, Zhang X (2012). Development of cell-active N6-methyladenosine RNA demethylase FTO inhibitor. J Am Chem Soc.

[53] Yan F, Al-Kali A, Zhang Z, Liu J, Pang J, Zhao N (2018). A dynamic N(6)-methyladenosine methylome regulates intrinsic and acquired resistance to tyrosine kinase inhibitors. Cell Res.

[54] Huang Y, Su R, Sheng Y, Dong L, Dong Z, Xu H (2019). Small-molecule targeting of oncogenic FTO emia. Cancer cell.

[55] Yankova E, Blackaby W, Albertella M, Rak J, De Braekeleer E, Tsagkogeorga G (2021). Small-molecule inhibition of METTL3 as a strategy against myeloid leukaemia. Nature.

[56] Ying Y, Ma X, Fang J, Chen S, Wang W, Li J (2021). EGR2-mediated regulation of m(6)A reader IGF2BP proteins drive RCC tumorigenesis and metastasis via enhancing S1PR3 mRNA stabilization. Cell death Dis.

[57] Li J, Xu X, Meng S, Liang Z, Wang X, Xu M (2017). MET/SMAD3/SNAIL circuit mediated by miR-323a-3p is involved in regulating epithelial-mesenchymal transition progression in bladder cancer. Cell death Dis.

[58] Xu X, Wu J, Li S, Hu Z, Xu X, Zhu Y (2014). Downregulation of microRNA-182-5p contributes to renal cell carcinoma proliferation via activating the AKT/FOXO3a signaling pathway. Mol cancer.

[59] Kausch I, Jiang H, Brocks C, Bruderek K, Krüger S, Sczakiel G (2004). Ki-67-directed antisense therapy in an orthotopic renal cell carcinoma model. Eur Urol.

