# Supplementary material for: FTO promotes clear cell renal cell carcinoma progression via upregulation of PDK1 through an m6A dependent pathway
Source: Cell Death Discov. 2022 Aug 12;8:356. doi: 10.1038/s41420-022-01151-w (PMC9374762; doi:10.1038/s41420-022-01151-w)
Supplement: Supplementary file 1 — Supplementary materials and methods [file 41420_2022_1151_MOESM1_ESM.docx]

**Supplementary materials and methods**

**m^6^A quantification**

The m^6^A quantification assay was performed according to the manual of the m^6^A RNA Methylation Quantification Kit (ab185912, Abcam). In brief, 200 ng total RNA samples of each group were added into the well plate. Then, the capture antibody and detection antibody were added followed by stop solution. The m^6^A content was quantified according to the absorbance value measured by a microplate reader at 450 nm.

**RNA stability assay**

Treated with actinomycin D (FD7007, Fude Biological Technology Company, 5 μg/mL) for 0, 2 and 4 h, the total RNA in YTHDF2-inhibited 786-O and Caki-1 cells was extracted and detected by RT-qPCR.
