# Supplementary material for: FTO promotes clear cell renal cell carcinoma progression via upregulation of PDK1 through an m6A dependent pathway
Source: Cell Death Discov. 2022 Aug 12;8:356. doi: 10.1038/s41420-022-01151-w (PMC9374762; doi:10.1038/s41420-022-01151-w)
Supplement: Supplementary file 2 — Supplementary Figure Legends [file 41420_2022_1151_MOESM2_ESM.docx]

**Supplementary Figure 1:** Cohorts from Oncomine database show FTO is notably up-regulated in various human cancers referred to normal tissues including ccRCC. A-E, FTO expression pattern in Finak Breast, Gaedcke Colorectal, Su Esophagus, Cho Gastric and Badea Pancreas cohorts. F, Expression profile of FTO in Jones Renal cohort.

**Supplementary Figure 2:** The expression pattern of FTO in kidney renal papillary cell carcinoma (KIRP) analyzed by the data of TCGA (A). The data of GSE16449 from the GEO database was utilized to validate the expression of FTO in ccRCC. ***p* < .01.

**Supplementary Figure 3:** The analysis of relative FTO (A) and PDK1 (B) protein level in HK-2 and RCC cell lines. All data are presented as the means ± SDs. **p* < .05, ***p* < .01 and ****p* < .001.

**Supplementary Figure 4:** The analysis of relative proteins level in FTO-silenced 786-O and Caki-1 cells. All data are presented as the means ± SDs. **p* < .05, ***p* < .01 and ****p* < .001.

**Supplementary Figure 5:** The knockdown efficacy of FTO with shRNAs in 786-O and Caki-1 cell lines was verified by Western blot assay. All data are presented as the means ± SDs. ***p* < .01 and ****p* < .001.

**Supplementary Figure 6:** H&E for muscle metastasis focus.

**Supplementary Figure 7:** Representative results of H&E for tumor focus and IHC for E-cadherin in the two groups.

**Supplementary Figure 8**: The relative m^6^A content of total RNA in FTO-silenced or overexpressed ccRCC cells. All data are presented as the means ± SDs. ***p* < .01 and ****p* < .001.

**Supplementary Figure 9:** Rescue experiments. A, Western blot assay shows the suppressed expression of PDK1 by siRNA is partially restored by FTO overexpression. B-C, Trans-well and colony formation assays show the inhibited migration and proliferation ability of 786-O cell line induced by knockdown of PDK1 is partially promoted by FTO overexpression. D, Western blot assay shows the suppressed expression of PDK1 by siRNA is partially restored by YTHDF2 knockdown. E-F, Trans-well and colony formation assays show the inhibited migration and proliferation ability of 786-O cell line induced by knockdown of PDK1 is partially promoted by YTHDF2 knockdown.

**Supplementary Figure 10:** The alteration of PDK1 mRNA detected by RT-qPCR when YTHDF2 (A) or YTHDF1 (B) knockdown in 786-O and Caki-1 cells. All data are presented as the means ± SDs. ***p* < .01 and ****p* < .001.

**Supplementary Figure 11:** The down-regulated expression profile of YTHDF2 in ccRCC vs normal tissues from the UALCAN database (A) and Kaplan-Meier curve for OS according to the relative expression of YTHDF2 in ccRCC from starBase (B). KIRC, kidney renal clear cell carcinoma. ****p* < .001.

**Supplementary Figure 12:** The analysis of relative proteins level in YTHDF2-overexpressed 786-O and Caki-1 cells. All data are presented as the means ± SDs. **p* < .05, ***p* < .01 and ****p* < .001.

**Supplementary Figure 13:** RIP-derived protein in 786-O and Caki-1 cell lines were measured by Western blot.

**Supplementary Figure 14:** Dual-luciferase reporter assay. Mutated m^6^A sites inhibited the binding of PDK1 to YTHDF2. (A) The schematic diagram of the dual luciferase vector pmirGlo and inserted wild-type and mutated sequence of m^6^A site. (B) Knockdown of YTHDF2 enhanced the luciferase activity of wild-type PDK1 but not of the mutated in 786-O and Caki-1 cells. **p* < .05.

**Supplementary Figure 15:** The analysis of relative proteins level in PDK1-silenced 786-O and Caki-1 cells. All data are presented as the means ± SDs. **p* < .05, ***p* < .01 and ****p* < .001.
