# Supplementary figures and images for: FTO promotes clear cell renal cell carcinoma progression via upregulation of PDK1 through an m6A dependent pathway
Source: Cell Death Discov. 2022 Aug 12;8:356. doi: 10.1038/s41420-022-01151-w (PMC9374762; doi:10.1038/s41420-022-01151-w)

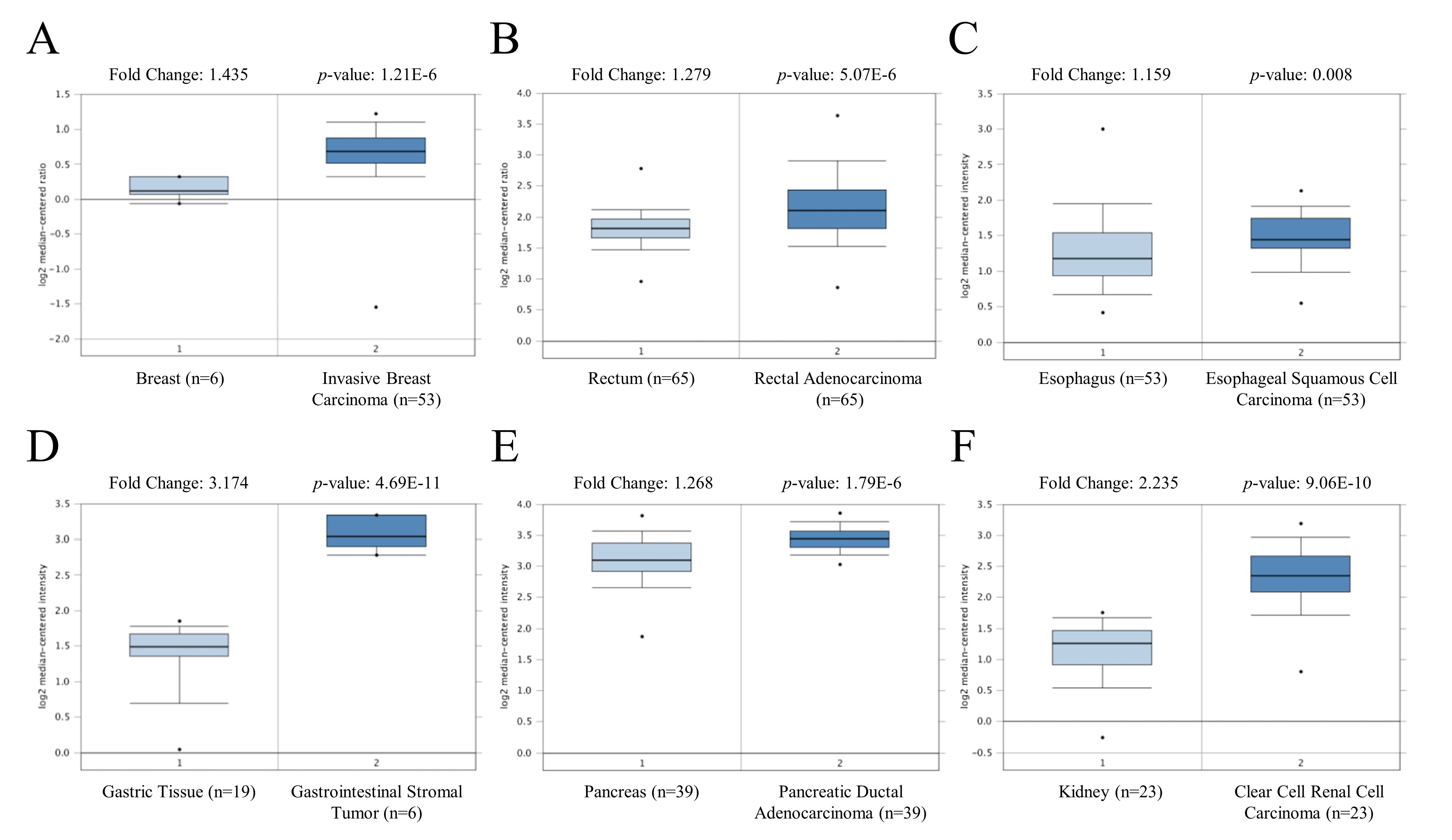

Supplement: Supplementary file 3 — Supplementary Figure 1 [file 41420_2022_1151_MOESM3_ESM.tif]

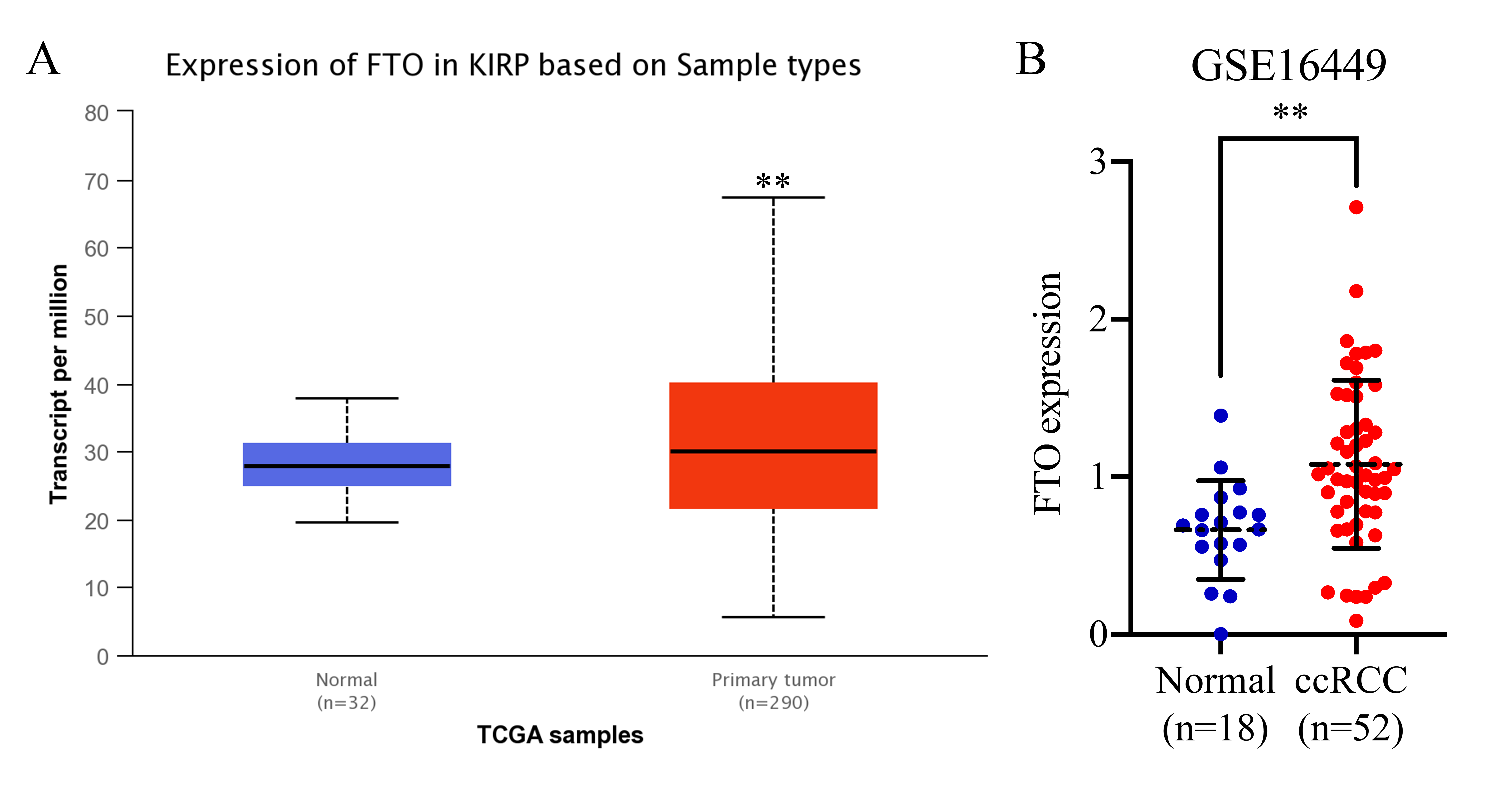

Supplement: Supplementary file 4 — Supplementary Figure 2 [file 41420_2022_1151_MOESM4_ESM.tif]

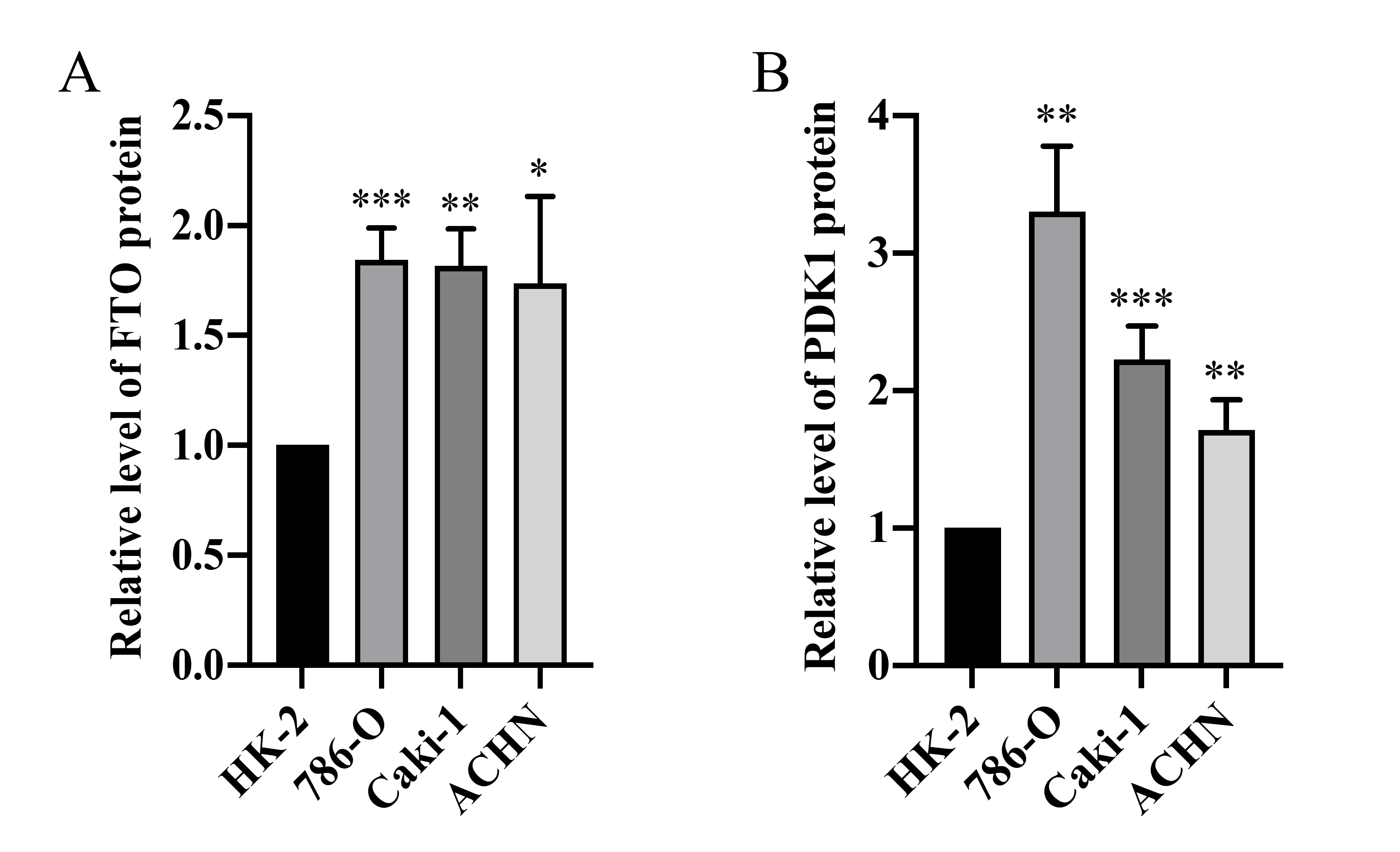

Supplement: Supplementary file 5 — Supplementary Figure 3 [file 41420_2022_1151_MOESM5_ESM.tif]

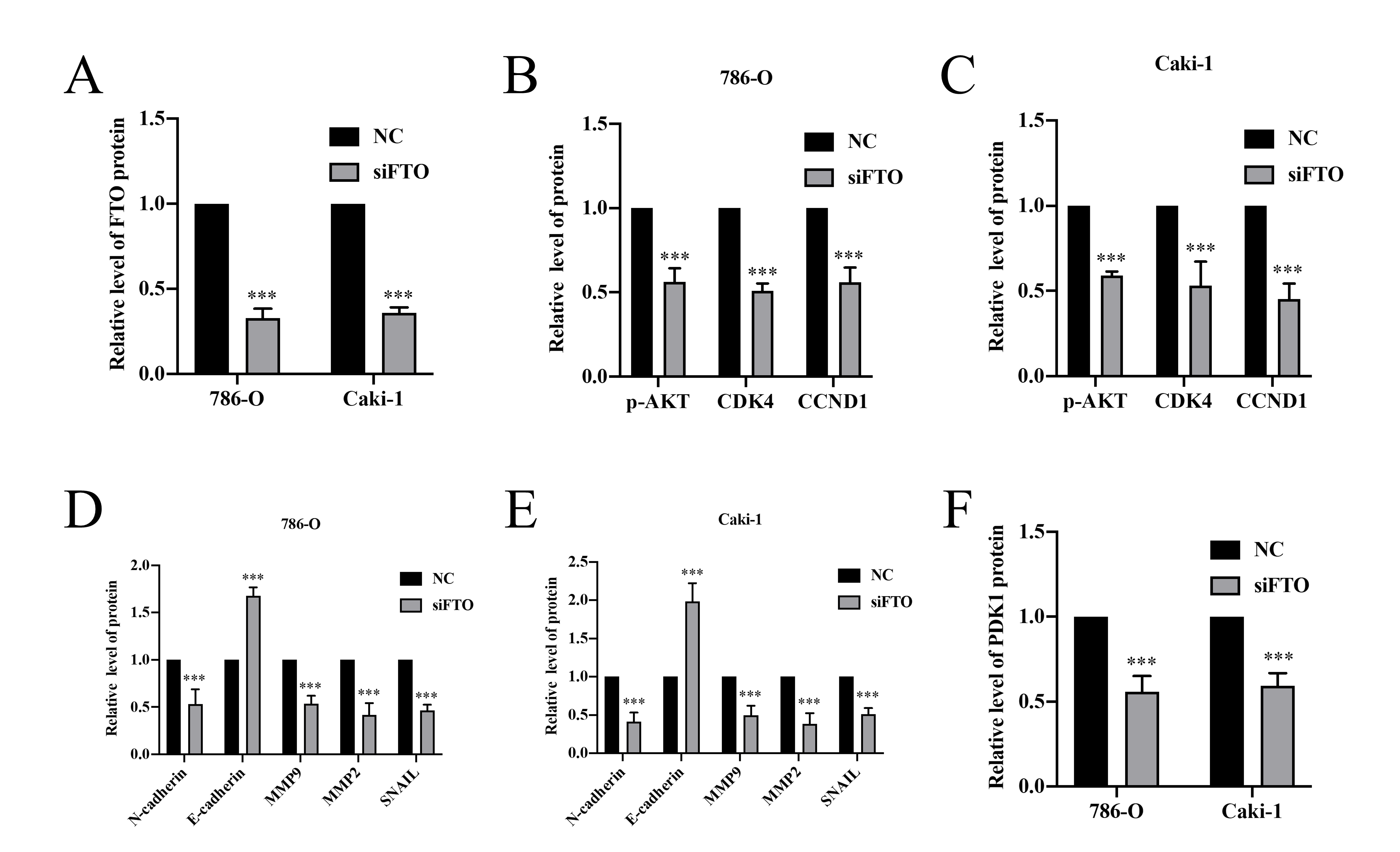

Supplement: Supplementary file 6 — Supplementary Figure 4 [file 41420_2022_1151_MOESM6_ESM.tif]

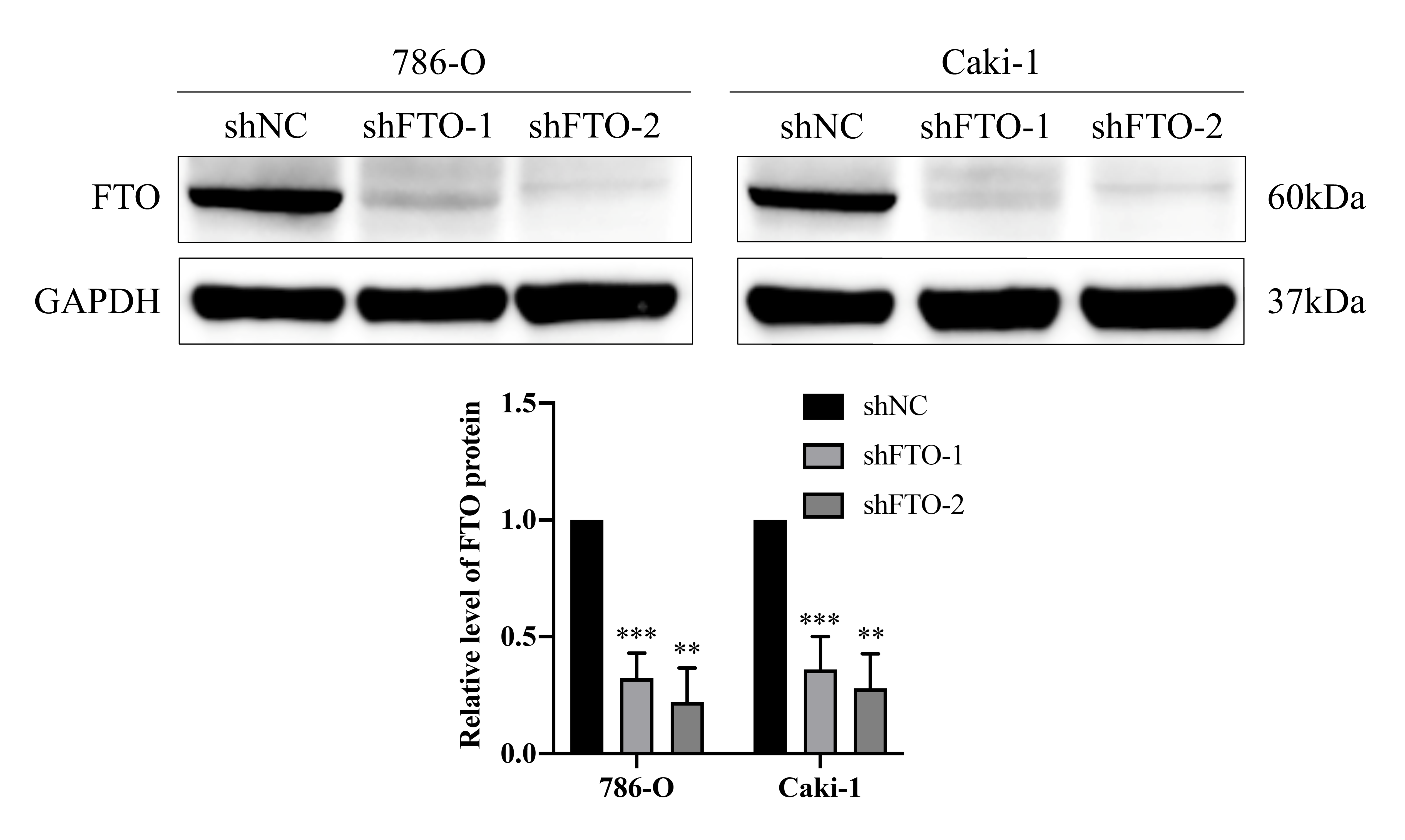

Supplement: Supplementary file 7 — Supplementary Figure 5 [file 41420_2022_1151_MOESM7_ESM.tif]

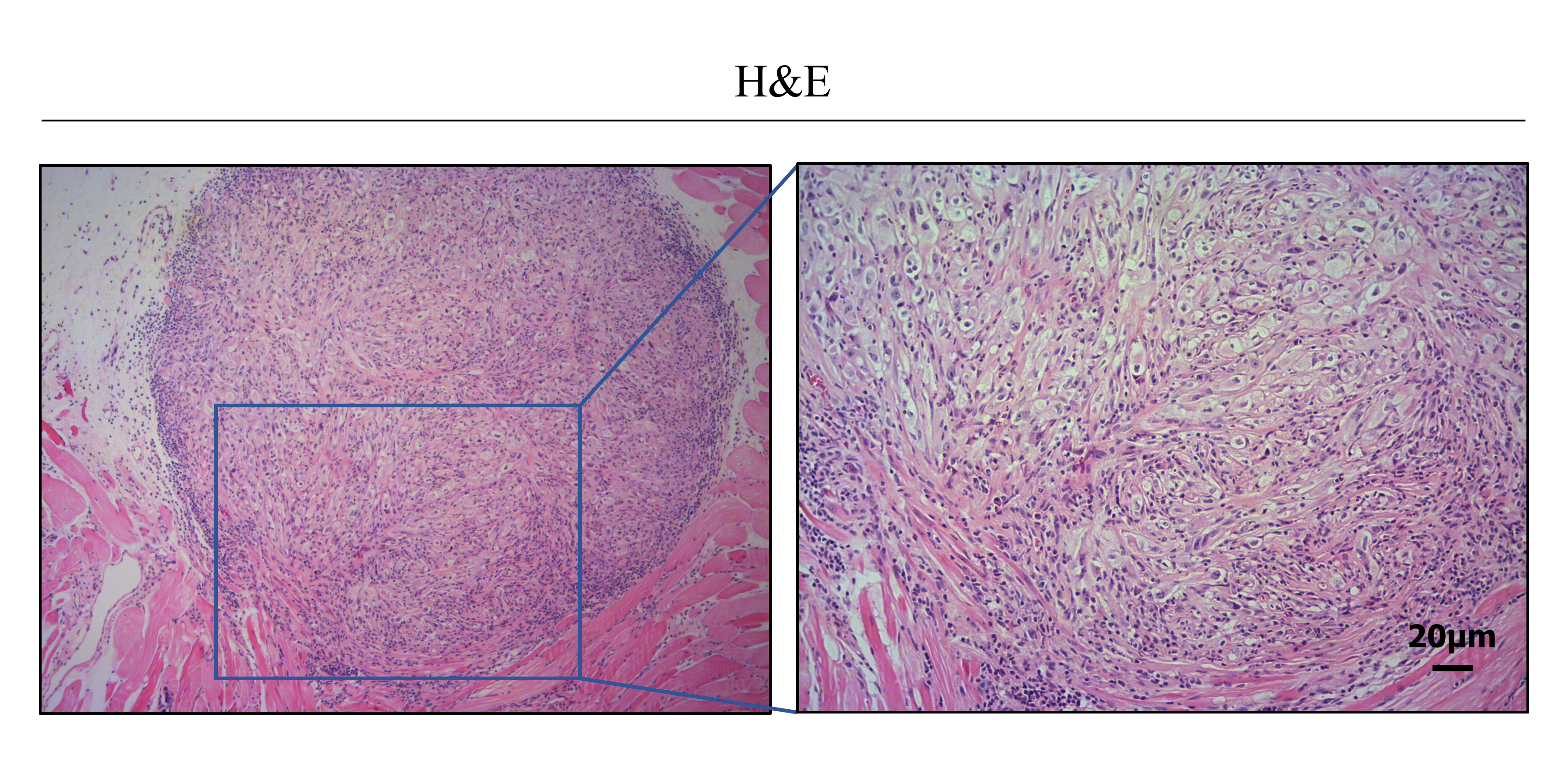

Supplement: Supplementary file 8 — Supplementary Figure 6 [file 41420_2022_1151_MOESM8_ESM.tif]

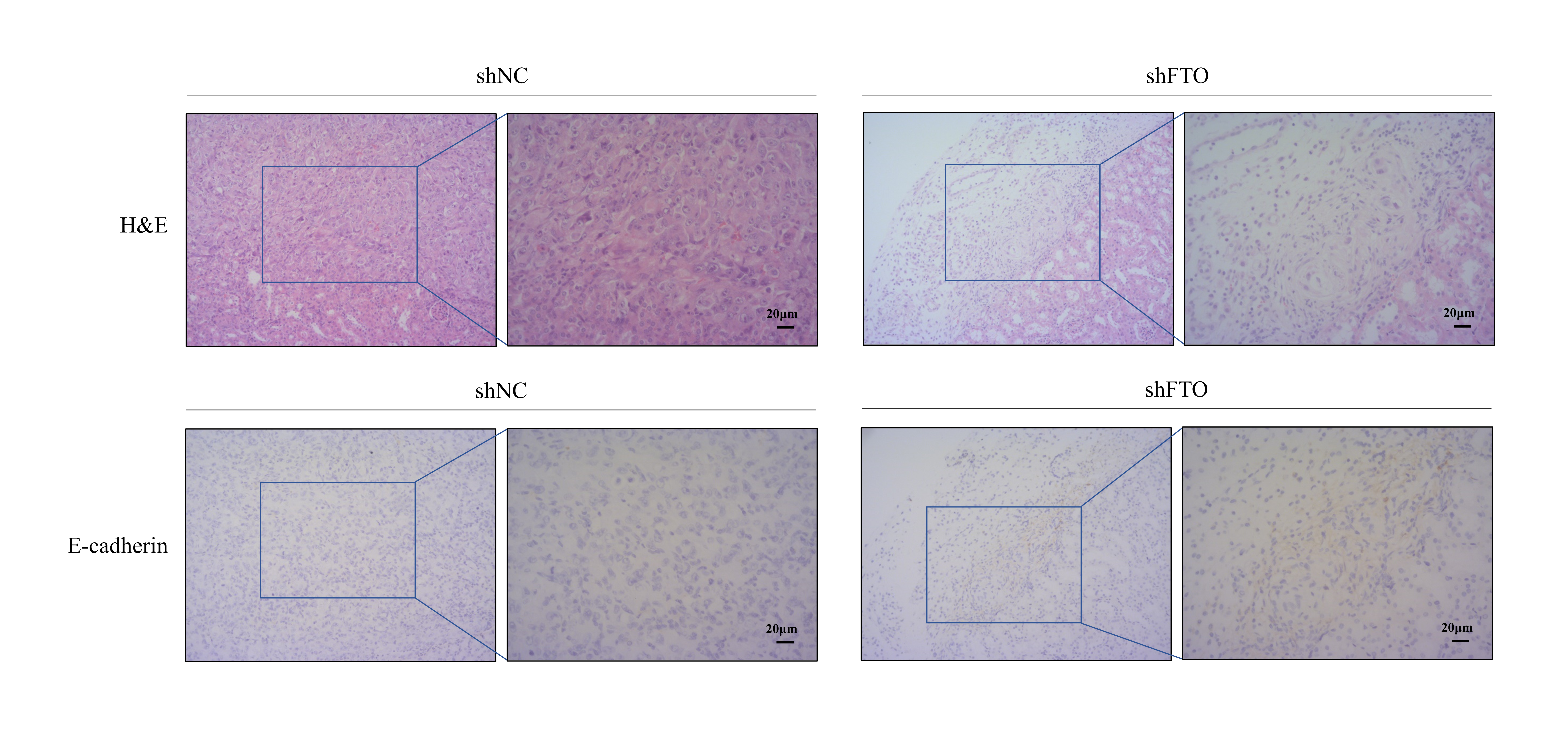

Supplement: Supplementary file 9 — Supplementary Figure 7 [file 41420_2022_1151_MOESM9_ESM.tif]

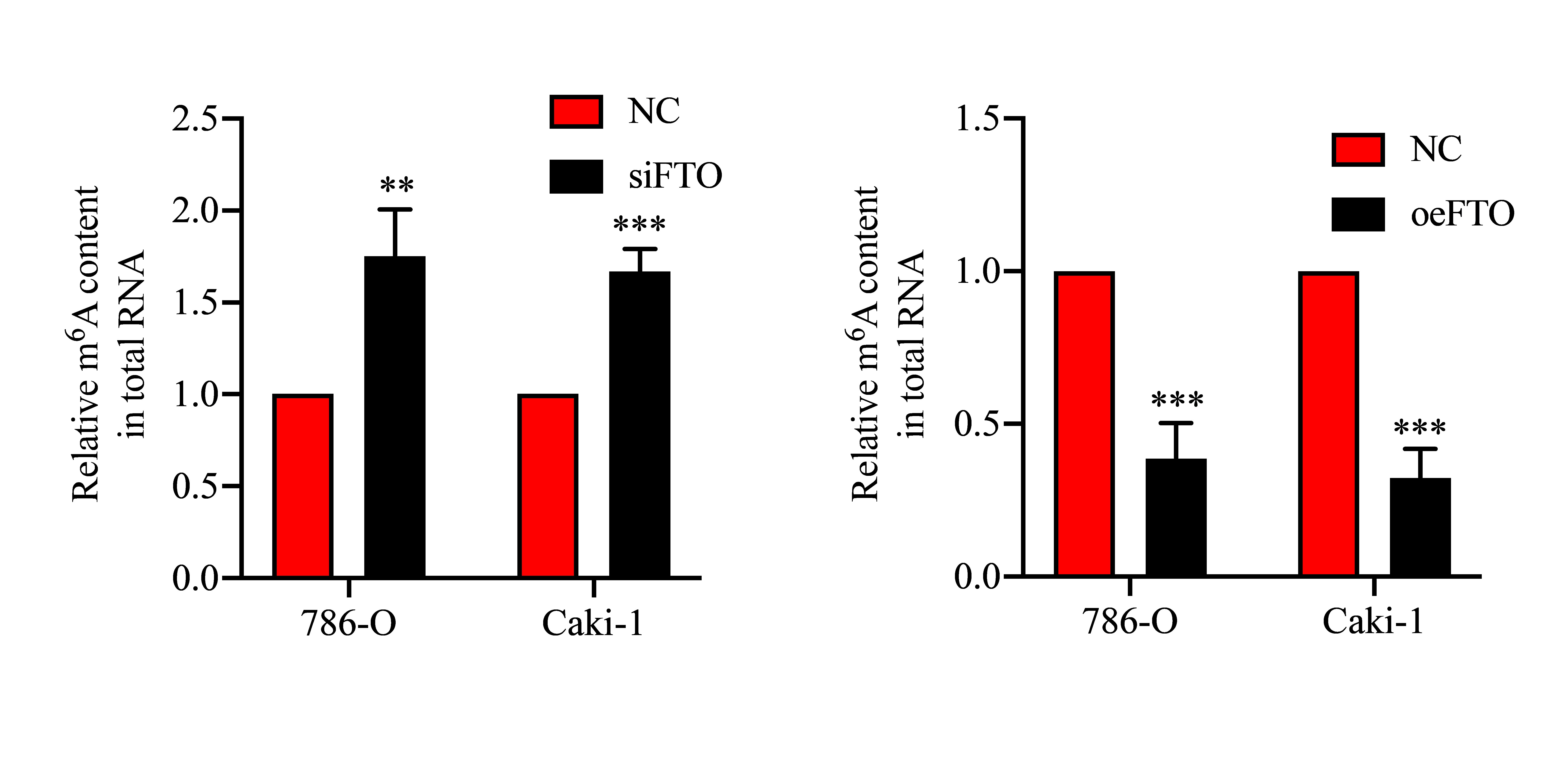

Supplement: Supplementary file 10 — Supplementary Figure 8 [file 41420_2022_1151_MOESM10_ESM.tif]

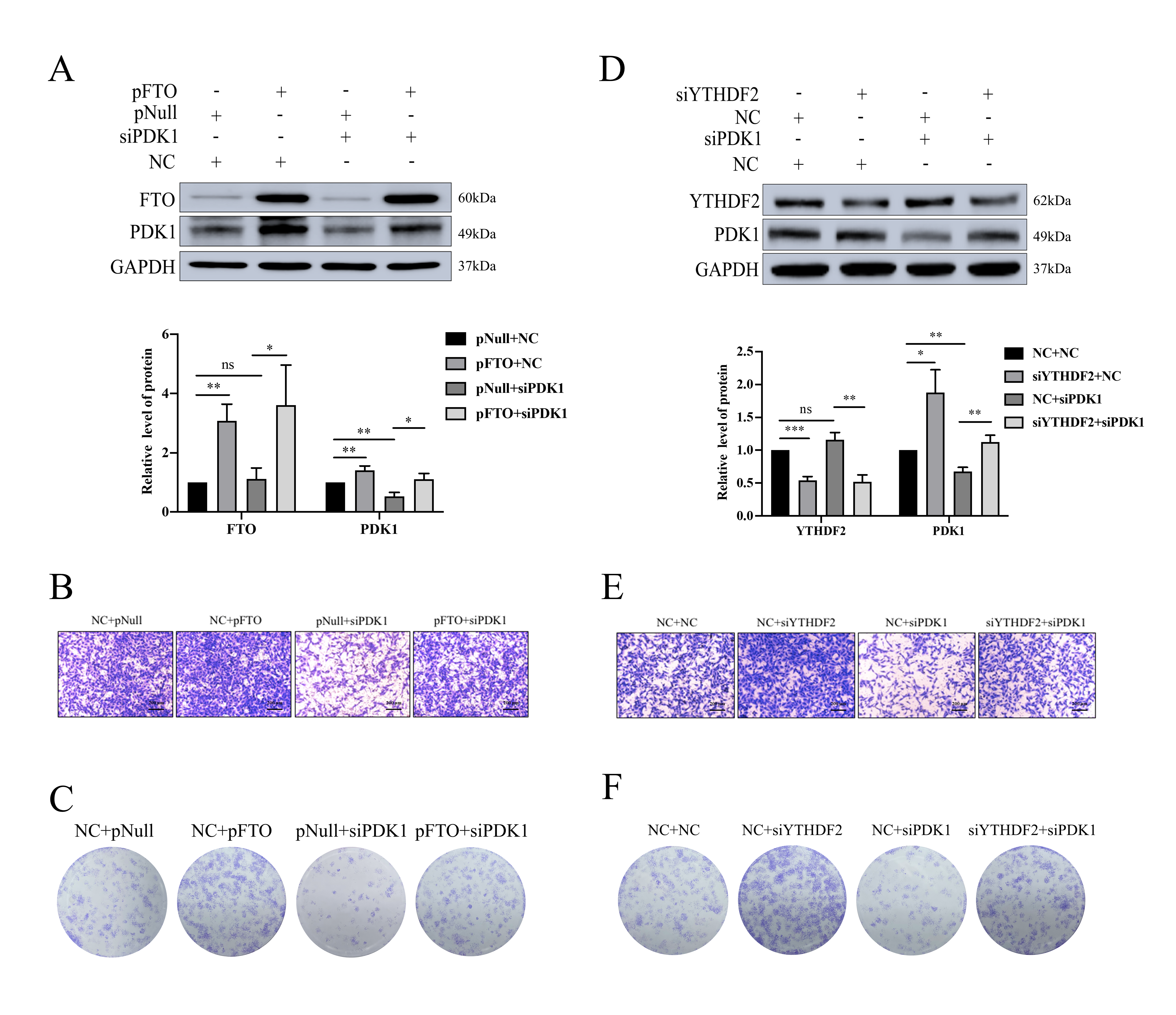

Supplement: Supplementary file 11 — Supplementary Figure 9 [file 41420_2022_1151_MOESM11_ESM.tif]

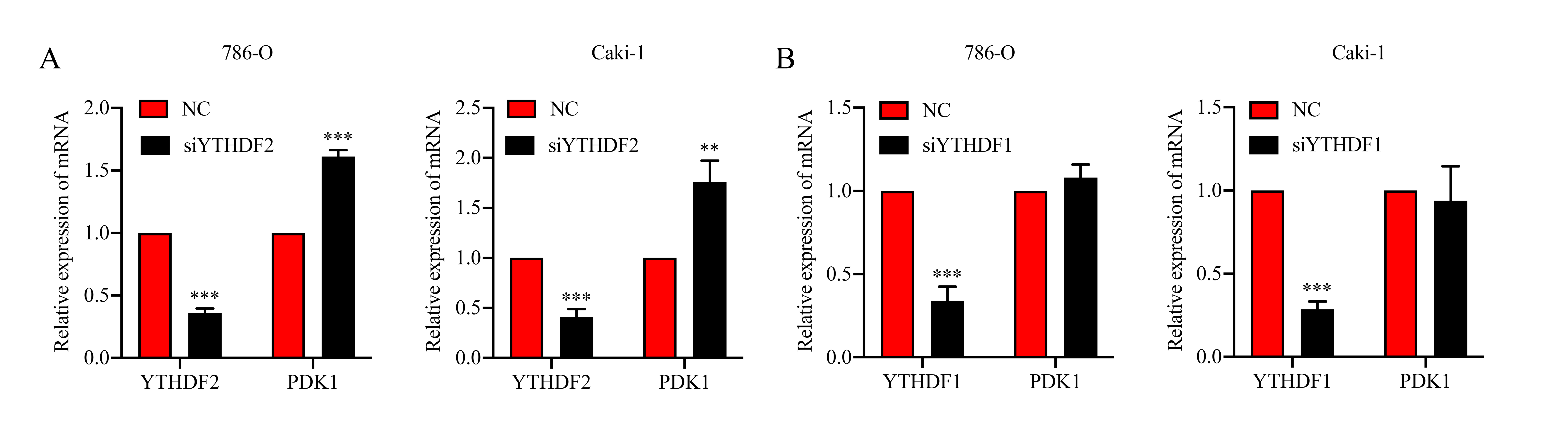

Supplement: Supplementary file 12 — Supplementary Figure 10 [file 41420_2022_1151_MOESM12_ESM.tif]

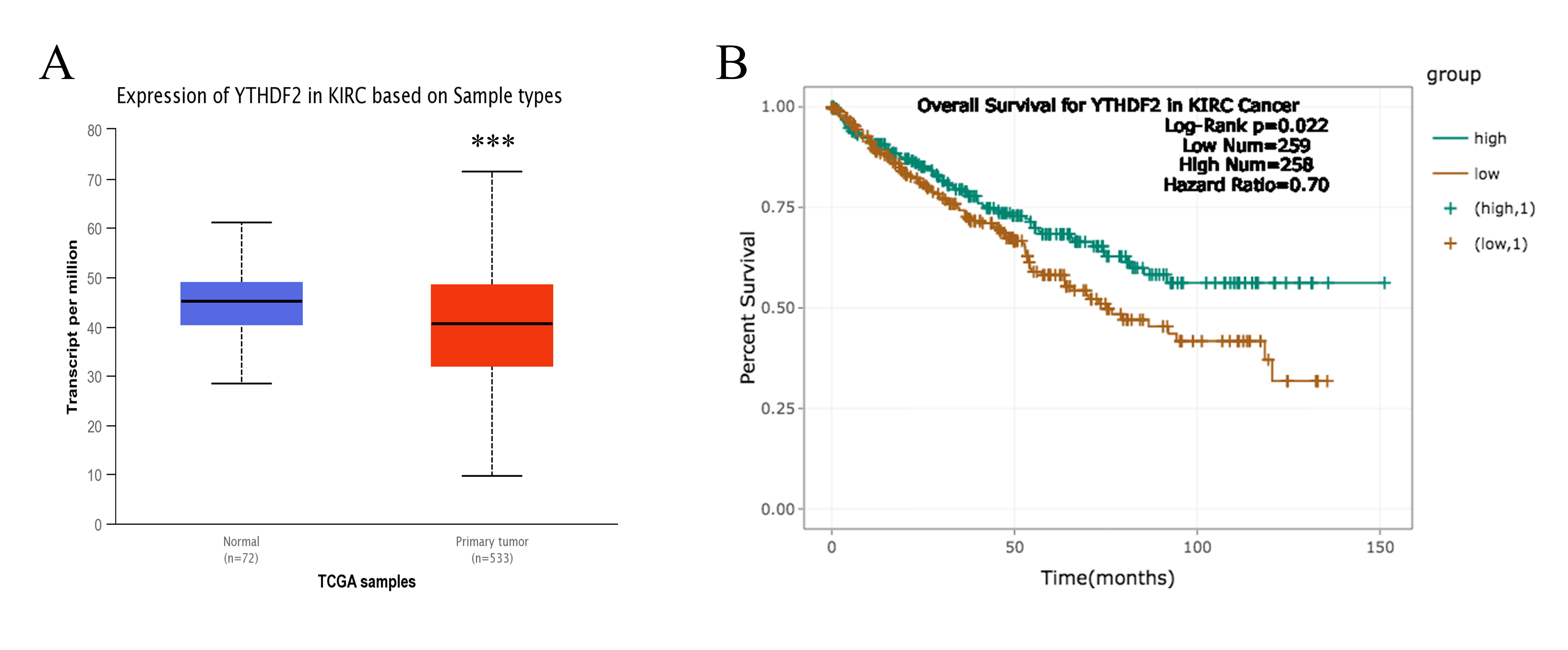

Supplement: Supplementary file 13 — Supplementary Figure 11 [file 41420_2022_1151_MOESM13_ESM.tif]

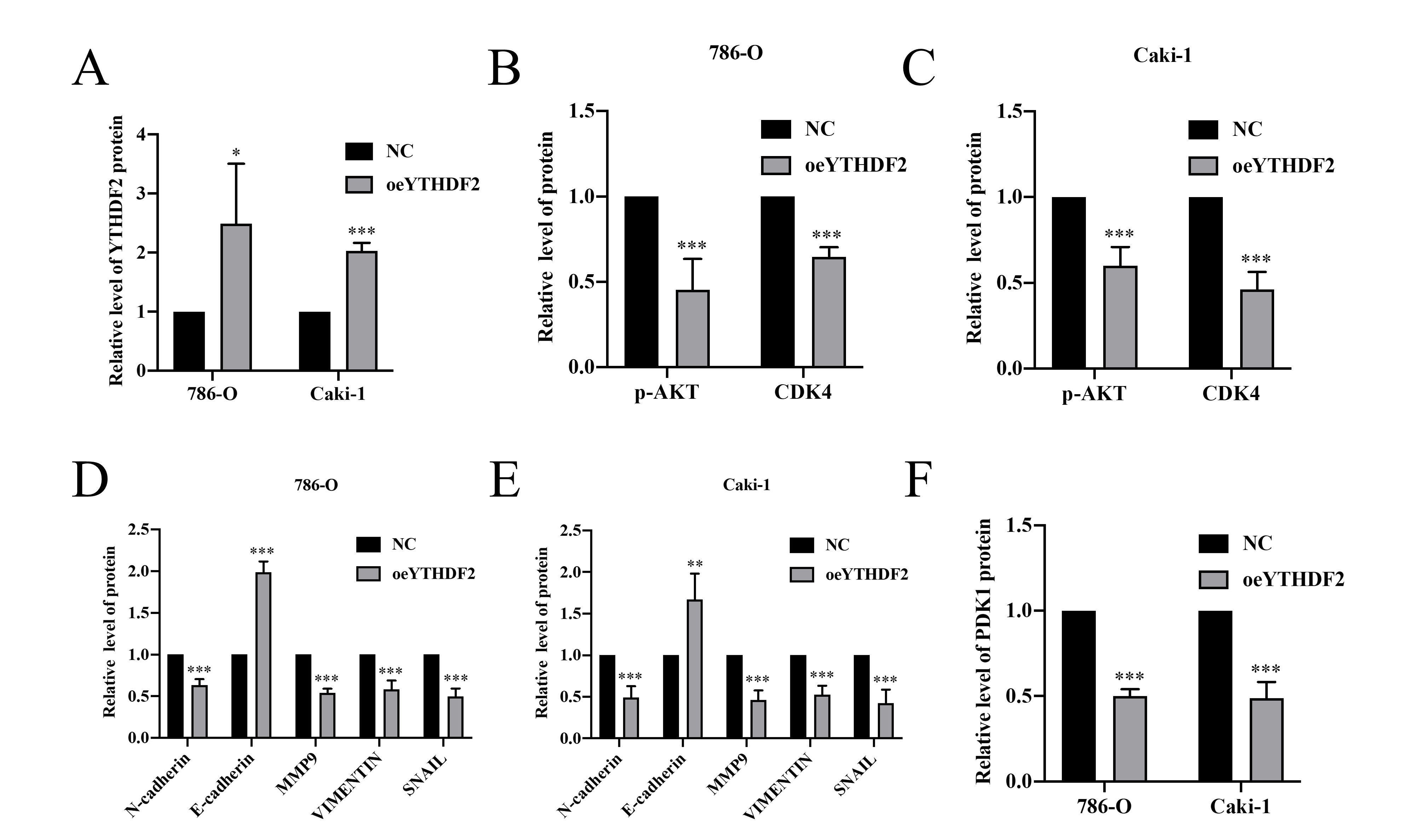

Supplement: Supplementary file 14 — Supplementary Figure 12 [file 41420_2022_1151_MOESM14_ESM.tif]

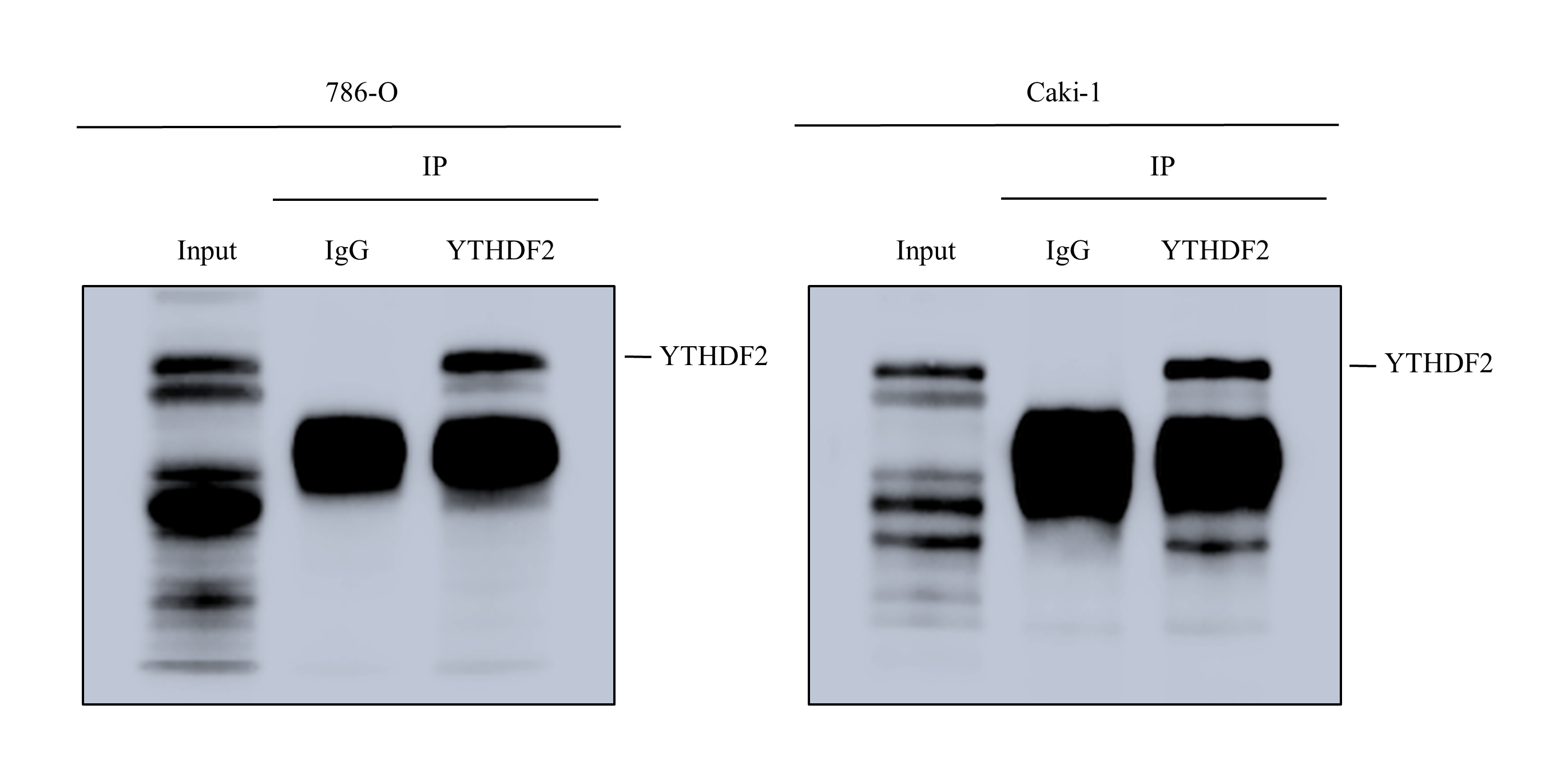

Supplement: Supplementary file 15 — Supplementary Figure 13 [file 41420_2022_1151_MOESM15_ESM.tif]

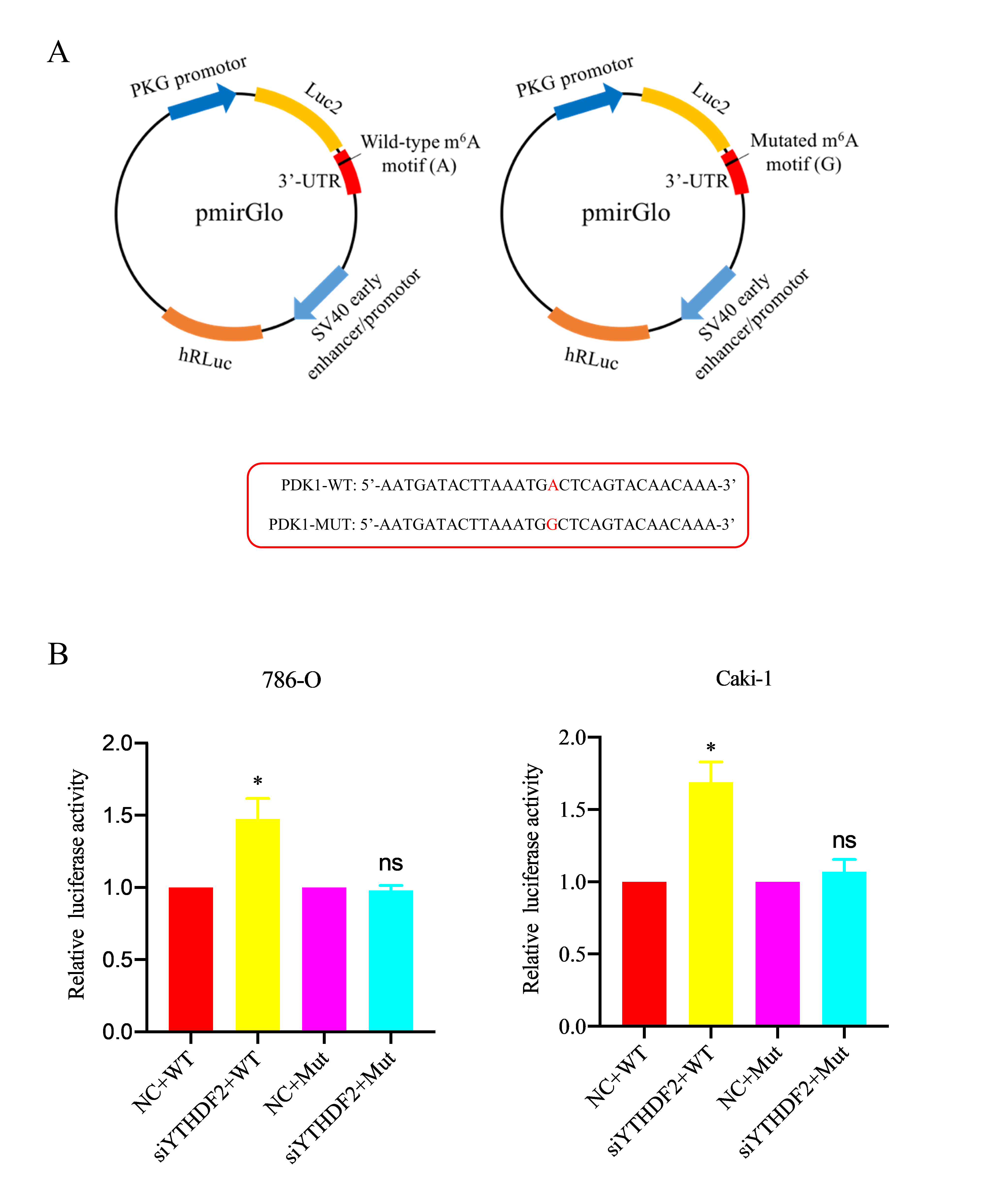

Supplement: Supplementary file 16 — Supplementary Figure 14 [file 41420_2022_1151_MOESM16_ESM.tif]

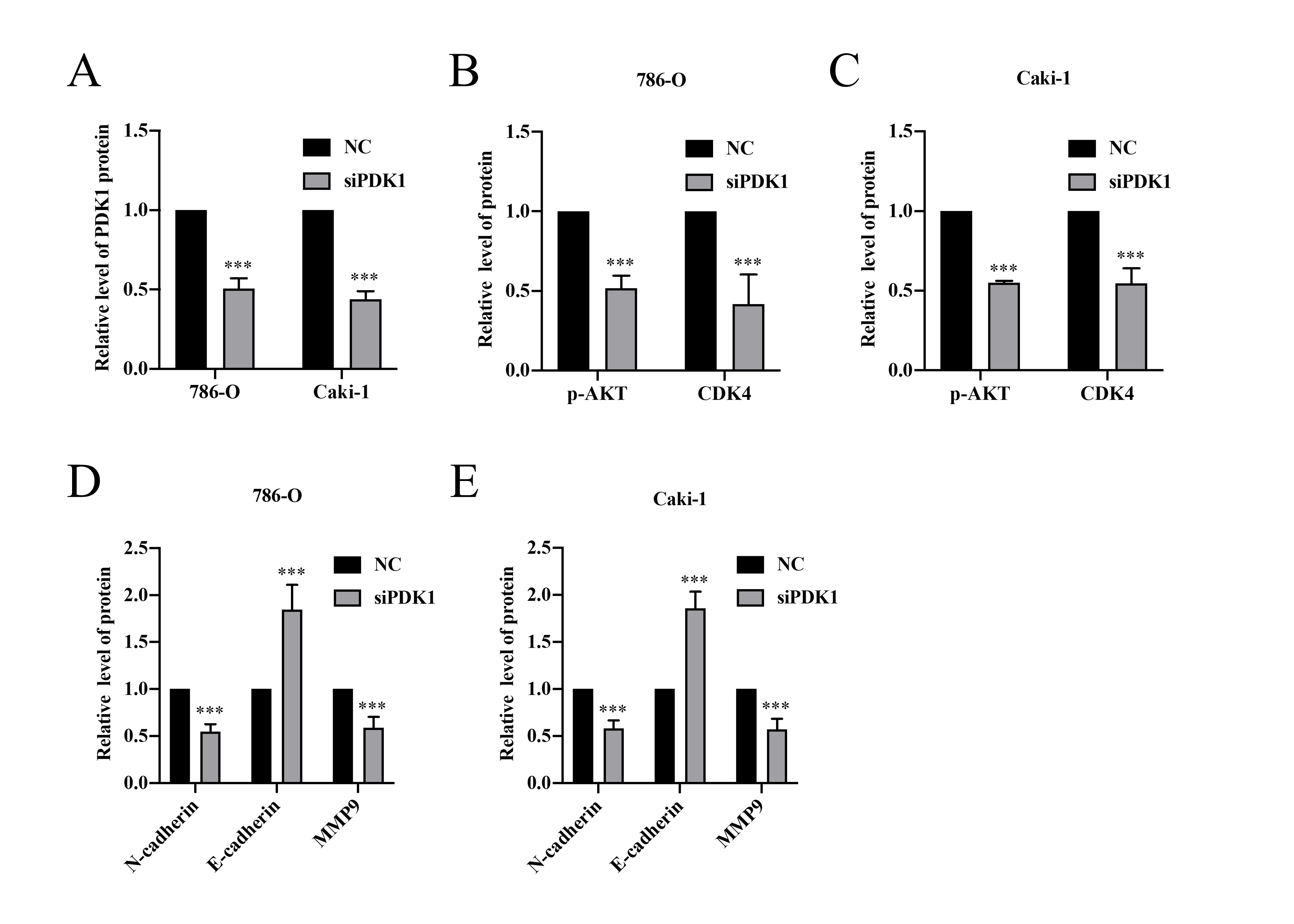

Supplement: Supplementary file 17 — Supplementary Figure 15 [file 41420_2022_1151_MOESM17_ESM.tif]
