# Supplementary material for: FTO promotes clear cell renal cell carcinoma progression via upregulation of PDK1 through an m6A dependent pathway
Source: Cell Death Discov. 2022 Aug 12;8:356. doi: 10.1038/s41420-022-01151-w (PMC9374762; doi:10.1038/s41420-022-01151-w)
Supplement: Supplementary file 20 — Supplementary Tables [file 41420_2022_1151_MOESM20_ESM.docx]

**Supplementary Table 1. The target sequences of siRNAs and shRNAs used in this study.**

| Name | Target Sequence (5’-3’) |
| --- | --- |
| siFTO-1 | CCTGAACACCAGGCTCTTT |
| siFTO-2 | GGATGACTCTCATCTCGAA |
| siFTO-3 | GTCACGAATTGCCCGAACA |
| siYTHDF2 | GCUCUGGAUAUAGUAGCAATT |
| siYTHDF1 | GGAUACAGUUCAUGACAAUTT |
| siPDK1-1 | GGAAGTCCATCTCATCGAA |
| siPDK1-2 | GAGTCGCATTTCAATTAGA |
| siPDK1-3 | GGAACACCATGCCAACAGA |
| shNC | TTCTCCGAACGTGTCACGT |
| shFTO-1 | GGCAGCTGAAATATCCTAAAC |
| shFTO-2 | GCTTTGGAAGAACTTGCTGCC |

**Supplementary Table 2. The primers used in this study.**

| Name^a^ | Sequence (5’-3’) |
| --- | --- |
| β-actin-F | ATCATGAAGTGTGACGTGGAC |
| β-actin-R | GACTCGTCATACTCCTGCTTG |
| FTO-F | ACTTGGCTCCCTTATCTGACC |
| FTO-R | TGTGCAGTGTGAGAAAGGCTT |
| PDK1-F | GAGAGCCACTATGGAACACCA |
| PDK1-R | GGAGGTCTCAACACGAGGT |
| YTHDF2-F | AGCCCCACTTCCTACCAGATG |
| YTHDF2-R | TGAGAACTGTTATTTCCCCATGC |
| YTHDF1-F | ACCTGTCCAGCTATTACCCG |
| YTHDF1-R | TGGTGAGGTATGGAATCGGAG |
| ^b^Me-PDK1-F | TGCTCAGTAAATACATAAGGAATAATG |
| ^b^Me-PDK1-R | TTTGCTAGTTTTGCACTGGTT |
| ^c^PDK1-Me-Wt-F | CAATGATACTTAAATGACTCAGTACAACAAAG |
| ^c^PDK1-Me-Wt-R | TCGACTTTGTTGTACTGAGTCATTTAAGTATCATTGAGCT |
| ^c^PDK1-Me-Mut-F | CAATGATACTTAAATGGCTCAGTACAACAAAG |
| ^c^PDK1-Me-Mut-R | TCGACTTTGTTGTACTGAGCCATTTAAGTATCATTGAGCT |

a: F, forward primer; R, reverse primer.

b: Primers used in MeRIP qRT-PCR.

c: Sequences inserted in pmirGlo vector between SacI and SalI sites for the dual luciferase activity assay.

**Supplementary Table 3. The associated antibodies used in this study.**

| Name | Catalog Number |
| --- | --- |
| anti-GAPDH | 60004-1-lg, Proteintech |
| anti-FTO | 14386S, Cell Signaling Technology |
| anti-CCND1 | 26939-1-AP, Proteintech |
| anti-CDK4 | 11026-1-AP, Proteintech |
| anti-AKT | 4691S, Cell Signaling Technology |
| anti-pAKT (S473) | 4058S, Cell Signaling Technology |
| anti-N-cadherin | 66219-1-lg, Proteintech |
| anti-E-cadherin^a^ | 20874-1-AP, Proteintech |
| anti-MMP2 | 10373-2-AP, Proteintech |
| anti-MMP9 | 10375-2-AP, Proteintech |
| anti-SNAIL | 13099-1-AP, Proteintech |
| anti-YTHDF2 | 24744-1-AP, Proteintech |
| anti-VIMENTIN | 10366-1-AP, Proteintech |
| anti-PDK1 | 10026-1-AP, Proteintech |
| anti-m^6^A | ab208577, Abcam |
| anti-Ki-67^a^ | 27309-1-AP, Proteintech |
| anti-FTO^a^ | 27226-1-AP, Proteintech |

a: Antibodies used for IHC.

**Supplementary Table 4. Histopathological types of RCC tissue samples (n=24).**

| Case NO. | Age | pT stage | Fuhrman grade | Histopathological type |
| --- | --- | --- | --- | --- |
| 1 | 55 | T1b | II | ccRCC |
| 2 | 55 | T2a | II | ccRCC |
| 3 | 36 | T1a | I | papillary RCC |
| 4 | 49 | T2a | II | ccRCC |
| 5 | 55 | T3a | II | ccRCC |
| 6 | 59 | T1b | I | ccRCC |
| 7 | 68 | T3a | II | ccRCC |
| 8 | 74 | T1b | I | ccRCC |
| 9 | 62 | T1b | II | ccRCC |
| 10 | 75 | T1a | I | ccRCC |
| 11 | 43 | T1a | I | ccRCC |
| 12 | 59 | T3b | III | ccRCC |
| 13 | 58 | T1b | I | ccRCC |
| 14 | 54 | T3a | I | ccRCC |
| 15 | 60 | T4 | II | ccRCC |
| 16 | 63 | T1b | II | ccRCC |
| 17 | 65 | T1b | I | ccRCC |
| 18 | 54 | T3a | II | ccRCC |
| 19 | 57 | T1a | I | ccRCC |
| 20 | 56 | T2a | III | ccRCC |
| 21 | 53 | T1b | I | ccRCC |
| 22 | 67 | T2a | I | ccRCC |
| 23 | 51 | T1b | II | ccRCC |
| 24 | 65 | T1b | I | ccRCC |
