# Supplementary material for: FTO promotes clear cell renal cell carcinoma progression via upregulation of PDK1 through an m6A dependent pathway
Source: Cell Death Discov. 2022 Aug 12;8:356. doi: 10.1038/s41420-022-01151-w (PMC9374762; doi:10.1038/s41420-022-01151-w)
Supplement: Supplementary file 21 — Original full length western blots [file 41420_2022_1151_MOESM21_ESM.docx]

Figure 1C FTO

Figure 1C GAPDH

Figure 2A 786-O FTO

Figure 2A 786-O GAPDH

Figure 2A Caki-1 FTO

Figure 2A Caki-1 GAPDH

Figure 2F 786-O pAKT

Figure 2F 786-O AKT

Figure 2F 786-O CDK4

Figure 2F 786-O CCND1

Figure 2F 786-O GAPDH

Figure 2F Caki-1 pAKT

Figure 2F Caki-1 AKT

Figure 2F Caki-1 CDK4

Figure 2F Caki-1 CCND1

Figure 2F Caki-1 GAPDH

Figure 2I 786-O N-cadherin

Figure 2I 786-O E-cadherin

Figure 2I 786-O MMP9

Figure 2I 786-O MMP2

Figure 2I 786-O SNAIL

Figure 2I 786-O GAPDH

Figure 2I Caki-1 N-cadherin

Figure 2I Caki-1 E-cadherin

Figure 2I Caki-1 MMP9

Figure 2I Caki-1 MMP2

Figure 2I Caki-1 SNAIL

Figure 2I Caki-1 GAPDH

Figure 4I 786-O PDK1

Figure 4I 786-O GAPDH

Figure 4I Caki-1 PDK1

Figure 4I Caki-1 GAPDH

Figure 5A 786-O YTHDF2

Figure 5A 786-O GAPDH

Figure 5A Caki-1 YTHDF2

Figure 5A Caki-1 GAPDH

Figure 5E 786-O pAKT

Figure 5E 786-O AKT

Figure 5E 786-O CDK4

Figure 5E 786-O GAPDH

Figure 5E Caki-1 pAKT

Figure 5E Caki-1 AKT

Figure 5E Caki-1 CDK4

Figure 5E Caki-1 GAPDH

Figure 5H 786-O N-cadherin

Figure 5H 786-O E-cadherin

Figure 5H 786-O MMP9

Figure 5H 786-O VIMENTIN

Figure 5H 786-O SNAIL

Figure 5H 786-O GAPDH

Figure 5H Caki-1 N-cadherin

Figure 5H Caki-1 E-cadherin

Figure 5H Caki-1 MMP9

Figure 5H Caki-1 VIMENTIN

Figure 5H Caki-1 SNAIL

Figure 5H Caki-1 GAPDH

Figure 5J 786-O PDK1

Figure 5J 786-O GAPDH

Figure 5J Caki-1 PDK1

Figure 5J Caki-1 GAPDH

Figure 6B PDK1

Figure 6B GAPDH

Figure 6C 786-O PDK1

Figure 6C 786-O GAPDH

Figure 6C Caki-1 PDK1

Figure 6C Caki-1 GAPDH

Figure 6G 786-O pAKT

Figure 6G 786-O AKT

Figure 6G 786-O CDK4

Figure 6G 786-O GAPDH

Figure 6G Caki-1 pAKT

Figure 6G Caki-1 AKT

Figure 6G Caki-1 CDK4

Figure 6G Caki-1 GAPDH

Figure 6J 786-O N-cadherin

Figure 6J 786-O E-cadherin

Figure 6J 786-O MMP9

Figure 6J 786-O GAPDH

Figure 6J Caki-1 N-cadherin

Figure 6J Caki-1 E-cadherin

Figure 6J Caki-1 MMP9

Figure 6J Caki-1 GAPDH

Supplementary Figure 5 FTO 786-O and Caki-1

Supplementary Figure 5 GAPDH 786-O and Caki-1

Supplementary Figure 5 FTO 786-O

Supplementary Figure 5 GAPDH 786-O

Supplementary Figure 5 FTO Caki-1

Supplementary Figure 5 GAPDH Caki-1

Supplementary Figure 9A FTO

Supplementary Figure 9A PDK1

Supplementary Figure 9A GAPDH

Supplementary Figure 9D YTHDF2

Supplementary Figure 9A PDK1

Supplementary Figure 9A GAPDH

Supplementary Figure 13 786-O

Supplementary Figure 13 Caki-1
